# Chronic skin and systemic inflammation modulated by S100A8 and S100A9 complexes

**DOI:** 10.1038/s41418-025-01504-9

**Published:** 2025-04-11

**Authors:** Marta Palomo-Irigoyen, Latifa Bakiri, Tim Hendrikx, Silvia Hayer, Johanna Schaffenrath, Stefanie Widder, Sandra Bachg, Jared Simmons, Richard L. Gallo, Philipp Starkl, Johannes Roth, Erwin F. Wagner

**Affiliations:** 1 Genes and Disease Laboratory, Department of Dermatology, Medical University of Vienna, Vienna, Austria; 2 Genes and Disease Laboratory, Department of Laboratory Medicine, Medical University of Vienna, Vienna, Austria; 3 Department of Laboratory Medicine, Medical University of Vienna, Vienna, Austria; 4 Department of Internal Medicine III, Division of Rheumatology, Medical University of Vienna, Vienna, Austria; 5 Division of Infection Biology, Department of Medicine I, Medical University of Vienna, Vienna, Austria; 6 Institute of Immunology, University of Münster, Münster, Germany; 7 Department of Dermatology, School of Medicine, University of California San Diego, La Jolla, CA USA

**Keywords:** Cell biology, Genetics, Immunology, Molecular biology, Diseases

## Abstract

Increased expression of the homodimeric S100A8 (A8) and S100A9 (A9) alarmins and their Calprotectin (CP) antimicrobial hetero-complex has been reported in Inflammatory Skin Diseases (ISDs) such as Atopic Dermatitis (AD), but the functional consequences of this increase are not known. We evaluated the cell- and tissue-specific functions of A8 and A9 in the local and the extra-cutaneous manifestations of ISD using genetically engineered mouse models. The genes encoding for the A9 or A8 proteins were inactivated in epidermal cells or neutrophils in the *JunB*^*∆ep*^ genetic mouse model for AD. Overall, epidermal inactivation of *A9* aggravated, while similar *A8* inactivation ameliorated experimental ISD. Epidermal differentiation and skin inflammation was also ameliorated when *A9* was inactivated in neutrophils or in all cells. However, complete *A9* knock-out was associated with worsened systemic effects, such as neutrophilic inflammation and bone loss. In addition, the distal phalanges of the digits displayed increased A8 protein expression, SA overgrowth and bone destruction. Epidermal *A8* inactivation ameliorated bone loss, but promoted bone destruction in the digits, likely through A8-positive neutrophilic infiltrates. These data show that site- and cell-type-specific A8 and A9 expression modulates chronic skin and systemic inflammation with distinct effects on the skin differentiation and on the musculoskeletal system. These findings pave the way for novel therapies targeting the divergent functions of A8 and A9 to restore epidermal homeostasis and prevent systemic complications.

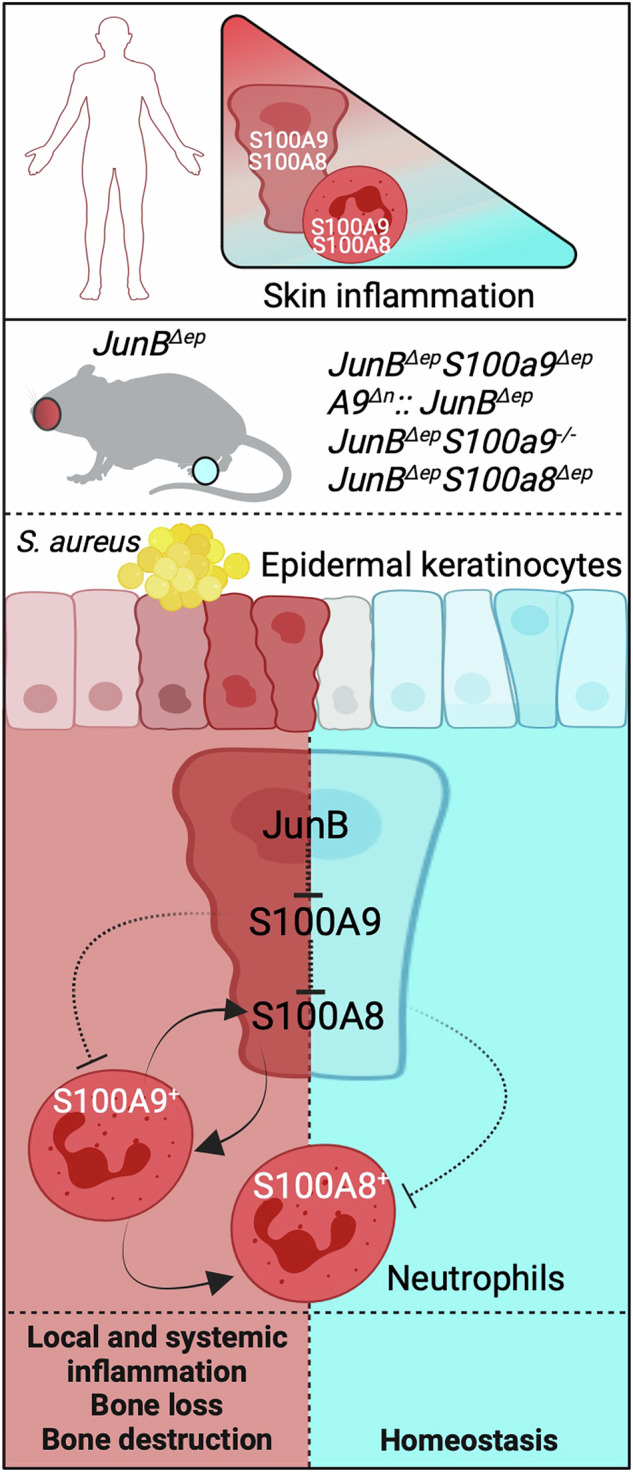

## Introduction

Skin diseases are among the most prevalent human health conditions affecting overall 900 million people worldwide [1, 2]. Inflammatory skin diseases (ISDs), such as Atopic Dermatitis (AD), are complex pathophysiological conditions where genetic susceptibility, environmental factors, epidermal barrier defects and immunological alterations are thought to play important roles [3–5]. AD accounts for the largest chronic ISD burden with a lifetime prevalence of up to 20% in children and 10% in adults [4]. AD is characterized by thickened epidermis due to excessive proliferation of basal keratinocytes, aberrant differentiation, suppression of terminal differentiation and disrupted filaggrin- and loricrin-expressing layers. This is likely responsible for epidermal barrier defects, immune cell infiltration into the skin, elevated Th2 and Th17 immune responses and increased immunoglobulin E (IgE) and itching, all AD hallmarks. Skin dysbiosis with *Staphylococcus aureus* (SA) colonization also frequently occurs in AD [3, 6, 7]. SA may even become invasive and cause serious but fortunately not common complications, such as endocarditis, osteomyelitis and septic arthritis [8, 9]. As broad-spectrum antibiotics failed to achieve a significant improvement in the cutaneous manifestations of AD, skin dysbiosis and SA colonization may not be a causative factor for the disease [10–12].

AD often extends beyond the skin with systemic inflammation propelled by a keratinocyte-immune-cell crosstalk and the release of chemokines and cytokines [13, 14]. Systemic inflammation may increase osteopenia and bone fracture risk in AD patients [15, 16]. Genetically engineered mouse models (GEMMs) of AD are powerful tools to study disease mechanisms across the entire body, in contrast to chemically-induced murine models, such as topical MC903 application or ovalbumin sensitization, which mostly cause a localized inflammation and fail to model extra-cutaneous disease manifestations [17, 18]. The JunB transcription factor, a component of the dimeric Activator Protein-1 (AP-1), plays a crucial role in the regulation of cytokine expression and immune response [19, 20]. Mice with genetic epidermal inactivation of *JunB* (*JunB*^*∆ep*^) develop distinctive AD hallmarks, including epidermal thickening, spontaneous SA colonization, immune cell infiltration into the skin and increased Th2 and Th17 inflammatory mediators [21, 22], such as IL-17A [21, 23], along with increased IgE, keratinocyte-secreted granulocyte-colony stimulating factor (G-CSF) [24] and IL-6 [25]. This leads to systemic inflammation with a myeloproliferative syndrome, body weight loss, splenomegaly and IL-17-dependent osteopenia [22–25]. Antibiotic treatment of diseased *JunB*^*∆ep*^ mice do not reduce skin inflammation, reminiscent of the human situation [21].

S100A8 (A8) and S100A9 (A9) homodimers and A8/A9 hetero-complexes, termed Calprotectin (CP), are implicated in ISDs [26–35], though their role as pro- or anti-inflammatory agents is not well-defined and the effects of cell-type-specific inactivation of either A8 or A9 in AD models have not been addressed. Keratinocytes and innate immune cells, such as neutrophils, are the major cell source of A8 and A9, which are not always co-expressed and can have distinct and even antagonistic effects. This is in part attributed to the co-existence of up to 3 different biological species, A8 and A9 homodimers and CP heterodimers or tetramers with differential binding abilities to receptors that include Toll-like receptor 4 (TLR4), receptor for advanced glycation end products (RAGE) and CD69. In addition, A8, A9 and CP can act intracellularly by modulating the cytoskeleton, in the nucleus through chromatin binding and extracellularly as damage-associated molecular pattern proteins (DAMPs) contributing to inflammation [26, 30, 36], while CP is antimicrobial as it chelates metal ions essential for bacteria [37].

A8- and A9-containing complexes likely have tissue- and organ-specific functions. *A9* knock-out alleviates skin and joint inflammation in a GEMM for psoriasis (Ps) [26], while skin-specific *A9* inactivation worsened Ps-like disease [27]. We also reported increased *A8* and *A9* expression in lesional skin homogenates of *JunB*^*∆ep*^ mice [21, 23]. However, the cell-specific involvement of A8, A9 and CP in ISD remain to be determined.

Here we show using novel GEMMs with cell-specific gene inactivation of *A9* or *A8* in *JunB*^*∆ep*^ mice that epidermal A9 restricts, while A8 enhances skin and systemic manifestations including bone loss. We unravel cell-, tissue- and site-specific divergent functions for A8 and A9, modulating local skin homeostasis and SA colonization, systemic inflammation-driven bone loss and SA-associated bone destruction in the digits.

## Results

### Increased *A8* and *A9* expression in epidermal and myeloid cells in human and murine skin inflammation

**Fig. 1:**
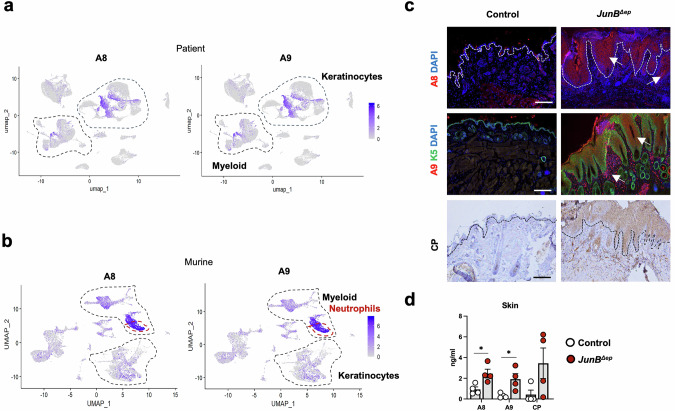
Increased *A8* and *A9* expression in epidermal and myeloid cells in human and murine skin inflammation. **a** *A8* and *A9* expression overlaid on UMAP of scRNA-seq analysis of 46,917 human skin cells. **b** Mouse *A8* and *A9* expression overlaid on UMAP of scRNA-seq analysis in skin from BALB/c mice, mock- or topically-treated with SA for 48 h or MC903-treated for 14 days followed by topical SA. **c** Representative images of skin sections from 6-months-old control and *JunB*^*∆ep*^ mice. A8 or A9 (red) immunofluorescence (IF) co-stained with Keratin 5 (K5) (green). Nuclei are stained with DAPI (blue) (top and middle panel). White arrows indicate A8- (top) or A9-positive cells (middle)]. Immunohistochemistry (IHC) for CP (bottom panel). A white (top panels) or black line (bottom panel) indicates the limits of epidermis. Scale bars, 200μm. **d** A8 dimers, A9 dimers and CP in skin lysates of 2 months-old *JunB*^*∆ep*^ mice and control littermates (ELISA). Dot plots represent mean ± SEM. *p ≤ 0.05. Unpaired 2-tailed Student’s t-test with Welch’s correction was applied.

Publicly available single-cell RNA-seq (scRNAseq) data from healthy individuals and lesional skin of AD patients (GSE153760) were analyzed for *A8* and *A9* expression [38]. Data from skin suction blistering, sampling skin cells and interstitial fluid, or conventional biopsies were plotted by Uniform Manifold Approximation and Projection (UMAP) (Fig. S1a). *A8* and *A9* expression overlapped, and was mainly localized in keratinocytes and to a lesser extent in myeloid cells of AD blister and biopsy samples (Fig. 1a, Fig. S1a–c). scRNAseq data from acute murine skin inflammation induced by topical application of MC903 and SA infection or SA alone (DRA015287) [38] indicated that either treatment increased *A8* and *A9* expression in most skin cell populations compared to mock-treated control mice (Fig. 1b, Fig. S1d–f). Myeloid cells, specifically neutrophils, expressed high levels of *A8* and *A9* mRNA in this acute model, unlike human AD samples, where keratinocytes were the major source of A8 and A9. In contrast, high protein abundance of A8, A9 and CP is observed in keratinocytes and infiltrating immune cells in the lesional skin of the *JunB*^*∆ep*^ mouse model of AD (Fig. 1c). Furthermore, A8, A9 and CP are increased in the skin and serum of *JunB*^*∆ep*^ mice as early as 2 months of age, before macroscopic skin lesions are visible (Fig. 1d, Fig. S1g, h). These data suggest a cell-specific contribution of A8 and A9 to the local and systemic manifestations of AD and lend sufficient support to the utilization of the *JunB*^*∆ep*^ model to dissect the cell-specific functional involvement of these proteins in ISD.

### Genetic inactivation of *A9* in epidermal cells aggravates skin disease in *JunB*^*∆ep*^ mice

**Fig. 2:**
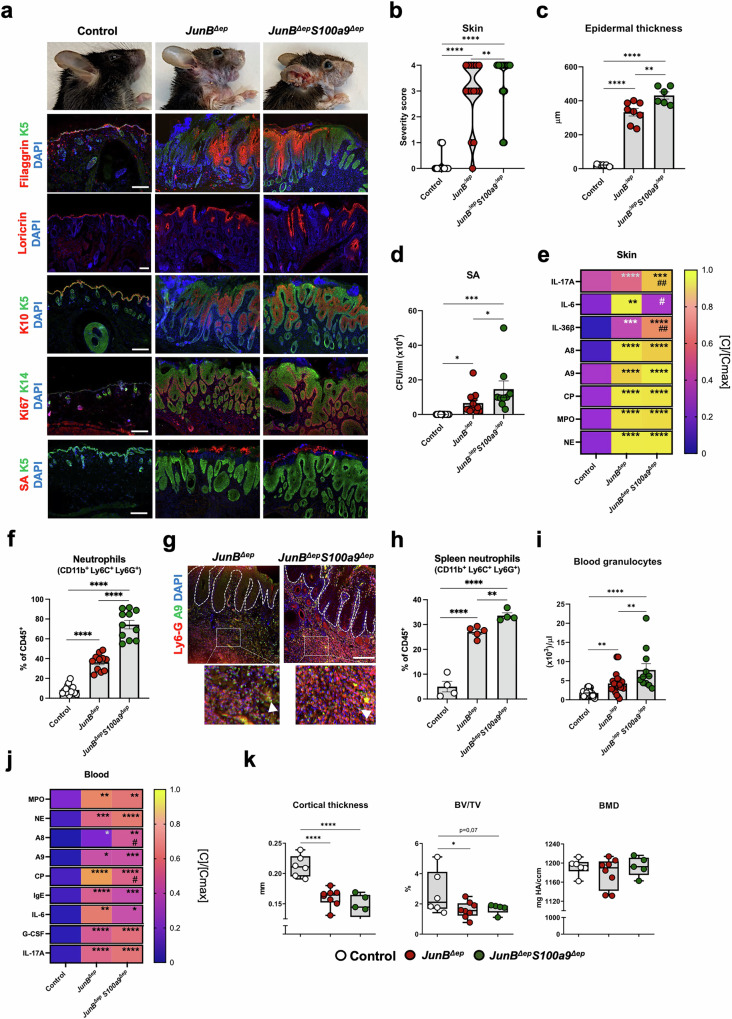
Genetic inactivation of *A9* in epidermal cells aggravates skin disease in *JunB*^*∆ep*^ mice. **a** Representative pictures of control, *JunB*^*∆ep*^ and *JunB*^*∆ep*^*S100a9*^*∆ep*^ mice and IF images of filaggrin (red), loricrin (red), keratin 10 (K10) (red), Ki67 (red) and *Staphylococcus aureus* (SA, red) with K5 (green) or K14 (green), as indicated. Nuclei are stained with DAPI. Scale bars, 200 μm. **b** Skin disease severity scoring from 0 (no lesions) to 4 (severe lesions in the face and ventral skin) of control, *JunB*^*∆ep*^ and *JunB*^*∆ep*^*S100a9*^*∆ep*^ mice. **c** Epidermal thickness (µm) measured on facial skin sections of mutant mice. Each dot represents the mean of 4 measurements for a single mouse. **d** SA colony-forming units (CFUs) in the skin of control, *JunB*^*∆ep*^ and *JunB*^*∆ep*^*S100a9*^*∆ep*^ mice. **e** Protein levels of IL-17A, IL-6, IL-36β, A8, A9, CP, MPO and NE in skin lysates from control (n = 4), *JunB*^*∆ep*^ (n = 4 < 7) and *JunB*^*∆ep*^*S100a9*^*∆ep*^ (n = 8) mice normalized ([C]/[Cmax]) by row. The maximum absolute value ([Cmax]) in ng/ml is IL-17A: 1.88, IL-6: 1.79, IL-36β: 2.63, A8: 5.62, A9: 2.88, CP: 7.46, MPO: 19.98, NE: 2.36. **f** Flow cytometric analysis of neutrophils (CD11b^+^, Ly6C^+^ and Ly6G^+)^ in the skin of *JunB*^*∆ep*^ and *JunB*^*∆ep*^*S100a9*^*∆ep*^ mice, shown as percentage (%) of CD45^+^ cells. **g** Representative IF images of neutrophils (Ly6G^+^) in red and A9-positive cells (green) in the skin of *JunB*^*∆ep*^ and *JunB*^*∆ep*^*S100a9*^*∆ep*^ mice, as indicated with white arrows. Nuclei are stained with DAPI. Scale bars, 200 μm**. h** Flow cytometric analysis of spleen neutrophils (CD11b^+^, Ly6C^+^ and Ly6G^+)^ in indicated mice, shown as percentage (%) of CD45^+^ cells. **i** Blood granulocyte counts in the indicated mice. **j** Protein levels of circulating MPO, NE, A8, A9, CP, IgE, IL-6, G-CSF, IL-17A in the serum of control (n = 7 < 10), *JunB*^*∆ep*^ (n = 6 < 9) and *JunB*^*∆ep*^*S100a9*^*∆ep*^ (n = 8 < 13) mice normalized ([C]/[Cmax]) by row. The maximum absolute value ([Cmax]) in ng/ml is MPO: 30.68, NE: 3.22, A8: 1.00, A9: 0.73, CP: 37.95, IgE: 883.33, IL-6: 0.02, G-CSF: 5.70, IL-17A: 0.26. **k** Quantification of the bone parameters cortical thickness, BV/TV and BMD in the indicated mice. Dot plots represent mean ± SEM. *p ≤ 0.05, **p ≤ 0.01, ***p ≤ 0.001, ****p ≤ 0.0001. Heat maps represent [C]/[Cmax] means, scaled by row. *p ≤ 0.05, **p ≤ 0.01, ***p ≤ 0.001, ****p ≤ 0.0001 compared to control mice and ^#^*p* ≤ 0.05, ^##^*p* ≤ 0.01 compared to *JunB*^*∆ep*^ mice. One-way ANOVA with Fishers’ LSD test was used for grouped statistical analysis. Unpaired 2-tailed Student’s t-test with Welch’s correction was applied to compare statistical difference between 2 groups (gray stars).

Genetic inactivation of *A9* in K5-positive (K5^+^) epidermal cells (*JunB*^*∆ep*^*S100a9*^*∆ep*^) led to a more severe skin disease (Fig. 2a, b). Worsened epidermal barrier alterations with disrupted filaggrin and loricrin expression were observed in *JunB*^*∆ep*^*S100a9*^*∆ep*^ mice together with increased K10^+^ differentiating and Ki67^+^ proliferating keratinocytes (Fig. 2a), thickening of the epidermis at the affected sites in the snout and ventral skin (Fig. 2a, c, Fig. S2a) and augmented SA colonization in the lesional skin (Fig. 2a, d). Epidermal *A9* gene inactivation in *JunB*^*∆ep*^ mice increased IL-17A and IL-36β, while reduced IL-6 protein levels were detected in total skin lysates compared to *JunB*^*∆ep*^ mice (Fig. 2e). High but comparable protein levels of A8, A9 and CP as well as the neutrophil-related inflammatory mediators Myeloperoxidase (MPO) and Neutrophil Elastase (NE) were measured in *JunB*^*∆ep*^*S100a9*^*∆ep*^ and *JunB*^*∆ep*^ mice (Fig. 2e). The absence of keratinocyte-derived A9 led to an almost 2-fold increase in CD45-positive (CD45^+^) immune cells and neutrophils infiltrating the lesional skin of *JunB*^*∆ep*^*S100a9*^*∆ep*^ mice (Fig. 2f, Fig. S2b). Neutrophils infiltrating the dermis expressed A9 in *JunB*^*∆ep*^ and *JunB*^*∆ep*^*S100a9*^*∆ep*^ mice, as shown by IF staining (Fig. 2g). CP-positive cells were also found in the dermis of *JunB*^*∆ep*^ and *JunB*^*∆ep*^*S100a9*^*∆ep*^ mice, whereas A8 was mostly detected in the epidermis of mutant mice (Fig. S2c). Neutrophils sorted from lesional skin of *JunB*^*∆ep*^ and *JunB*^*∆ep*^*S100a9*^*∆ep*^ mice expressed similar amounts of *A8* or *A9* mRNA (Fig. S2d). These data indicate that the majority of CP and A9 dimers in the skin of *JunB*^*∆ep*^*S100a9*^*∆ep*^ is derived from infiltrating neutrophils.

No differences in body weight loss were observed between the two mutant groups, but splenomegaly was more prominent in the absence of epidermal A9 (Fig. S2e) with increased splenic neutrophils compared to *JunB*^*∆ep*^ littermates (Fig. 2h). Bone marrow (BM) neutrophils (Fig. S2f) and blood granulocytes (Fig. 2i) were also higher in *JunB*^*∆ep*^*S100a9*^*∆ep*^ mutants, while BM CD45^+^ immune cells and blood lymphocytes were similar to *JunB*^*∆ep*^ mice (Fig. S2f, g). Serum A8 dimers were significantly increased while CP was decreased in *JunB*^*∆ep*^*S100a9*^*∆ep*^ mice, when compared to *JunB*^*∆ep*^, whereas A9, MPO, NE, IgE, IL-6, G-CSF and IL-17A, while elevated, protein levels were similar to those observed in *JunB*^*∆ep*^ littermates (Fig. 2j). Consistent with comparable circulating IL-17A, the reduction in bone cortical thickness and in bone volume to tissue volume (BV/TV) measured by μCT in the tibiae was similar between the two mutant groups, while bone mineral density (BMD) was comparable between all groups (Fig. 2k). Overall, these data indicate that *A9* gene inactivation in keratinocytes worsens the skin manifestations in *JunB*^*∆ep*^ mice, increases local and circulating neutrophil numbers, but except for splenomegaly, has little impact on the systemic manifestations of the disease and in particular bone loss.

### Loss of neutrophil-derived A9 ameliorates skin disease in *JunB*^*∆ep*^ mice

**Fig. 3:**
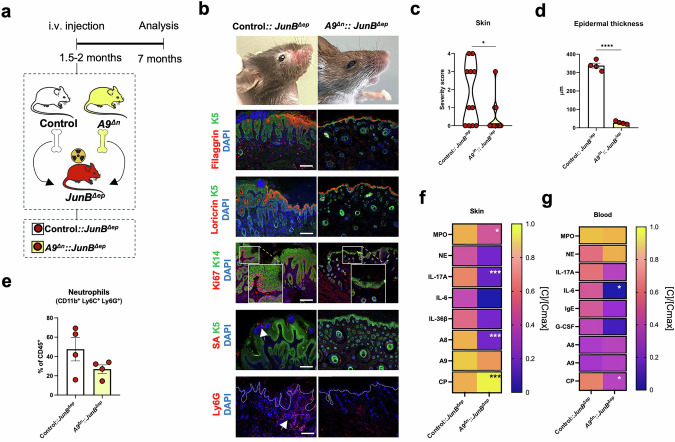
Loss of A9 in neutrophils ameliorates skin inflammation in *JunB*^*∆ep*^ mice. **a** Scheme of bone marrow transplantation (BMT) experiments. **b** Representative pictures of control::*JunB*^*∆ep*^ and *A9*^*∆n*^*::JunB*^*∆ep*^ bone marrow chimeric mice and IF staining of filaggrin (red), loricrin (red), Ki67 (red) and SA (red), as indicated with a white arrow, and co-stained with K5 (green) or K14 (green) in skin sections of control::*JunB*^*∆ep*^ and *A9*^*∆n*^*::JunB*^*∆ep*^ chimeric mice and Ly6G^+^ neutrophils (red), as indicated with the white arrow. Nuclei are stained with DAPI. Scale bars, 200μm. **c** Skin disease severity scoring from 0 (no lesions) to 4 (severe lesions in the face and ventral skin) of the indicated mice. **d** Epidermal thickness (µm) measured on facial skin sections of control::*JunB*^*∆ep*^ and *A9*^*∆n*^*::JunB*^*∆ep*^ mice. Each dot represents the mean of 4 measurements for a single mouse. **e** Flow cytometric analysis of neutrophils (CD11b^+^, Ly6C^+^ and Ly6G^+^) in the skin of control::*JunB*^*∆ep*^ and *A9*^*∆n*^*::JunB*^*∆ep*^ mice, shown as percentage (%) of CD45^+^ cells. **f** Protein levels of MPO, NE, IL-17A, IL-6, IL-36β cytokines and A8, A9 and CP alarmins in skin homogenates of control::*JunB*^*∆ep*^ (n = 5) and *A9*^*∆n*^*::JunB*^*∆ep*^ (n = 5) chimeric mice normalized ([C]/[Cmax]) by row. The maximum absolute value ([Cmax]) in ng/ml is MPO: 13.98, NE: 3.41, IL-17A:1.51, IL-6: 4.94, IL-36β: 2.33, A8: 4.26, A9: 4.03, CP: 13.28. **g** Protein levels of circulating MPO, NE, IL-17A, IL-6, IgE, G-CSF, A8, A9 and CP in the serum of control::*JunB*^*∆ep*^ (n = 4 < 7) and *A9*^*∆n*^*::JunB*^*∆ep (n*^ = 3 < 7) chimeras normalized ([C]/[Cmax]) by row. The maximum absolute value ([Cmax]) in ng/ml is MPO: 62.86, NE: 6.023, IL-17A: 0.27, IL-6: 0.021, IgE: 4914.573, G-CSF: 28.80, A8: 0.78, A9: 0.97, CP: 22.09. Dot plots represent mean ± SEM. *p ≤ 0.05, **p ≤ 0.01, ***p ≤ 0.001, ****p ≤ 0.0001. Heat maps represent [C]/[Cmax] means, scaled by row, *p ≤ 0.05, **p ≤ 0.01, ***p ≤ 0.001. Student’s t-test with Welch’s correction was applied.

Mice with A9-deficient neutrophils (*A9*^*∆n*^) were generated using the myeloid/granulocyte-specific *Mrp8-Cre* allele [39] (Fig. S3a, b). Deletion of the *A9* floxed allele was confirmed in neutrophils sorted from the BM (Fig. S3c). *A9*^*∆n*^ mice were indistinguishable from control littermates with no differences in BM neutrophils (Fig. S3d), or in systemic parameters, such as body weight, spleen-to-body weight ratio (Fig. S3e) and blood cell counts (Fig. S3f). BM cells from *A9*^*∆n*^ mice or A9-proficient littermates were transplanted into lethally irradiated *JunB*^*∆ep*^ mice (Fig. 3a). Epidermal ISD manifestations were less severe in *JunB*^*∆ep*^ mice transplanted with *A9*^*∆n*^ BM (*A9*^*∆n*^::*JunB*^*∆ep*^) with, no visible macroscopic skin lesions, compared to *JunB*^*∆ep*^ mice that received BM with A9-proficient neutrophils (control*::JunB*^*∆ep*^) (Fig. 3b, c). Histology revealed a uniform expression of filaggrin and loricrin, less Ki67^+^ keratinocytes, undetectable SA and infiltrating neutrophils in the skin of *A9*^*∆n*^::*JunB*^*∆ep*^ mice with a marked reduction in epidermal thickness (Fig. 3b, d). The decrease in skin-infiltrating neutrophils was confirmed by flow cytometry (Fig. 3e). MPO, NE, IL-17A, IL-6 and IL-36β were decreased in skin lysates of *A9*^*∆n*^::*JunB*^*∆ep*^ mice compared to *JunB*^*∆ep*^, although only IL-17A and MPO reached statistical significance (Fig. 3f). Epidermal A8 and A9 were also diminished in *A9*^*∆n*^::*JunB*^*∆ep*^ skin sections (Fig. S3g) and although A9 dimers appeared marginally affected, lower levels of A8 dimers and higher levels of CP were measured in skin lysates from *A9*^*∆n*^::*JunB*^*∆ep*^ mice (Fig. 3f). Body weight loss and spleen-to-body weight ratio were comparable between the two groups (Fig. S3h). Circulating MPO, NE, IL-17A, IgE, G-CSF, A8 and A9 were slightly decreased in *A9*^*∆n*^::*JunB*^*∆ep*^ mice compared to *JunB*^*∆ep*^, while CP and IL-6 reached statistical significance (Fig. 3g). These results establish a pro-inflammatory function of A9 expression in neutrophils in AD-like disease, which contrasts with the role of A9 in epidermal cells and supports a cell-specific action of A9-containing complexes.

### Complete *A9* knock-out ameliorates skin inflammation, but aggravates systemic disease in *JunB*^*∆ep*^ mice

**Fig. 4:**
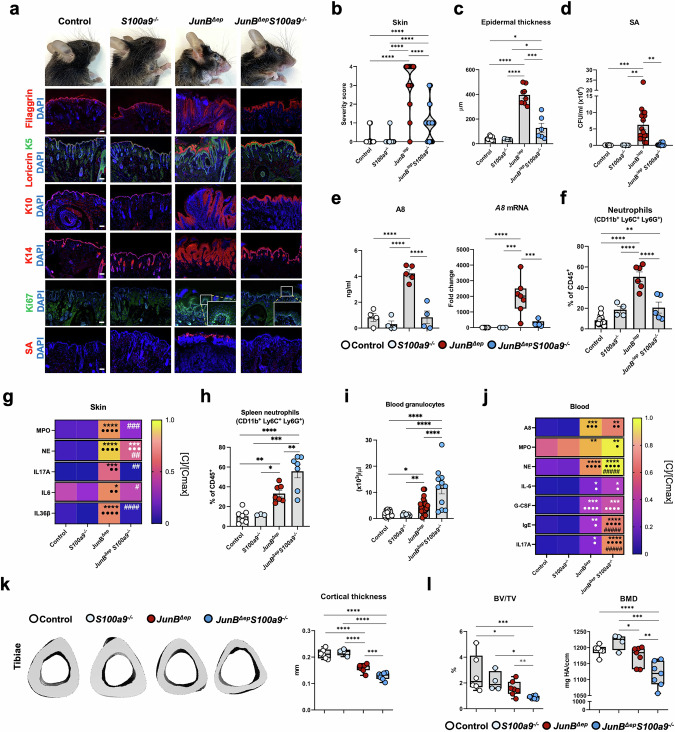
*A9* knock-out improves skin inflammation, but aggravates systemic disease in *JunB*^*∆ep*^ mice. **a** Representative pictures of control, *S100a9*^*−/−*^*, JunB*^*∆ep*^ and *JunB*^*∆ep*^*S100a9*^*−/−*^ mice and IF stainings of filaggrin (red), loricrin (red) co-stained with K5(green), and K10 (red), K14 (red), Ki67 (green) and SA (red). Nuclei are stained with DAPI. Scale bars, 100μm. **b** Skin disease severity scoring from 0 (no lesions) to 4 (severe lesions in the face and ventral skin) in the indicated mice. **c** Epidermal thickness (µm) measured on facial skin sections of mutant mice. Each dot represents the mean of 4 measurements for a single mouse. **d** SA CFUs in the skin of control, *S100a9*^*−/−*^*, JunB*^*∆ep*^ and *JunB*^*∆ep*^*S100a9*^*−/−*^ mice. **e** *A8* mRNA (left) and A8 protein levels (right) in skin lysates of indicated mice. **f** Flow cytometric analysis of neutrophils (CD11b^+^, Ly6C^+^ and Ly6G^+^) in the skin of control, *S100a9*^*−/−*^*, JunB*^*∆ep*^ and *JunB*^*∆ep*^*S100a9*^*−/−*^ mice, shown as percentage (%) of CD45^+^ cells. **g** Protein levels of MPO, NE, IL-17A, IL-6 and IL-36β cytokines in skin lysates of control (n = 4), *S100a9*^*−/−*^ (n = 4)*, JunB*^*∆ep*^ (n = 4) and *JunB*^*∆ep*^*S100a9*^*−/−*^ (n = 4 < 5) mice normalized ([C]/[Cmax]) by row. The maximum absolute value ([Cmax]) in ng/ml is MPO: 25.94, NE: 2.37, IL-17A: 0.62, IL-6: 2.02, IL-36β: 1.61. **h** Flow cytometric analysis of neutrophils (CD11b^+^, Ly6C^+^ and Ly6G^+)^ in the spleen, shown as percentage (%) of CD45^+^ cells in control, *S100a9*^*−/−*^*, JunB*^*∆ep*^ and *JunB*^*∆ep*^*S100a9*^*−/−*^ mice. **i** Granulocyte counts in the blood of control and mutant mice. **j** Protein levels of circulating A8, MPO, NE, IgE, IL-6, G-CSF and IL-17A in the serum of control (n = 4 < 8), *S100a9*^*−/−*^ (n = 4)*, JunB*^*∆ep*^ (n = 4 < 17) and *JunB*^*∆ep*^*S100a9*^*−/−*^ (n = 4 < 7) mice normalized ([C]/[Cmax]) by row. The maximum absolute value ([Cmax]) in ng/ml is A8: 0.09, MPO: 31.80, NE: 6.98, IgE: 1595.65, IL-6: 0.07, G-CSF: 8.50 and IL-17A: 0.78. **k** Representative μCT images of the tibiae (left) and quantification of the cortical thickness of the tibiae (right) of control, *JunB*^*∆ep*^; *S100a9*^*−/−*^ and *JunB*^*∆ep*^*S100a9*^*−/−*^ mice. **l** Quantification of BV/TV and BMD in the indicated mice. One-way ANOVA with Fishers’ LSD test was used for statistical grouped analysis, as well as unpaired 2-tailed Student’s t-test with Welch’s correction was applied to compare statistical difference between 2 groups (gray stars). Dot plots represent mean ± SEM. *p ≤ 0.05, **p ≤ 0.01, ***p ≤ 0.001, ****p ≤ 0.0001. Heat maps represent [C]/[Cmax] means, scaled by row, *p ≤ 0.05, **p ≤ 0.01, ***p ≤ 0.001, ****p ≤ 0.0001 compared to control mice, ^•^*p* ≤ 0.05 ^••^*p* ≤ 0.01, ^•••^*p* ≤ 0.001, ^••••^*p* ≤ 0.0001 compared to S100a9^−/−^ mice and ^#^*p* ≤ 0.05 ^##^*p* ≤ 0.01, ^###^*p* ≤ 0.001, ^####^*p* ≤ 0.0001 compared to *JunB*^*∆ep*^ mice.

We generated *JunB*^*∆ep*^*S100a9*^*−/−*^ mice lacking A9 and CP in all cells of the body and therefore only producing A8 dimers. A marked improvement of the ISD phenotype was observed in the snout and ventral skin of *JunB*^*∆ep*^*S100a9*^*−/−*^ mice, with reduced macroscopic skin lesions compared to *JunB*^*∆ep*^ mice (Fig. 4a, b, Fig. S4a). Histologically, the epidermis was thinner (Fig. 4a, c, Fig. S4a), as were the layers of K5^+^, K14^+^ and Ki67^+^ proliferative and K10^+^ differentiating keratinocytes (Fig. 4a, Fig. S4a). The expression pattern of filaggrin and loricrin was also restored to control levels (Fig. 4a) and skin SA colonization abolished (Fig. 4a, d, Fig. S4b). IHC and ELISA revealed decreased A8 protein expression and A8 dimers that was confirmed by qPCR (Fig. 4e, Fig. S4c). The lack of A9-containing complexes resulted in an almost complete normalization of the number of skin-infiltrating immune cells and neutrophils (Fig. 4f, Fig. S4d). Consistently, MPO and NE were reduced in *JunB*^*∆ep*^*S100a9*^*−/−*^ total skin lysates, along with IL-17A, IL-6 and IL-36β, reaching the values measured in healthy controls (Fig. 4g). However, and in contrast to the beneficial effect observed in the snout and ventral skin, *JunB*^*∆ep*^*S100a9*^*−/−*^ mice had increased body weight loss and splenomegaly (Fig. S4e), pointing to a more severe systemic disease. Spleen neutrophils were increased in *JunB*^*∆ep*^*S100a9*^*−/−*^ mice (Fig. 4h), along with BM CD45^+^ immune cells and, although not statistically significant, BM neutrophils (Fig. S4f). Blood granulocytes were also increased (Fig. 4i), whereas lymphocytes were unaffected (Fig. S4g). While A8 dimers, IL-6, G-CSF and MPO were elevated to a similar extent in the serum of *JunB*^*∆ep*^ and *JunB*^*∆ep*^*S100a9*^*−/*−^ mice (Fig. 4j), NE, IgE and IL-17A were further increased (Fig. 4j). Consistent with higher IL17A, cortical thickness, BV/TV and even BMD were decreased in the tibiae of *JunB*^*∆ep*^*S100a9*^*−/−*^ mice (Fig. 4k, l). These data indicate that the pro-inflammatory function of A9-expressing neutrophils is dominant over the anti-inflammatory role of A9-expressing keratinocytes in modulating the skin but also the systemic manifestations of AD-like disease.

### Digit swelling and local bone destruction with SA colonization in *JunB*^*∆ep*^*S100a9*^*−/−*^ mice

**Fig. 5:**
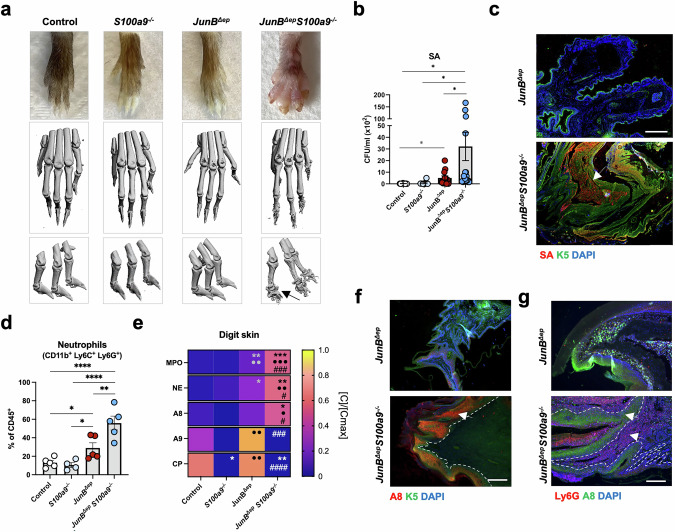
Digit swelling and local bone destruction with SA colonization in *JunB*^*∆ep*^*S100a9*^*−/−*^ mice. **a** Representative images of hind paws of the indicated mice (top panel) and μCT images of the digits (middle and bottom panels). The black arrow indicates bone destruction in the distal phalanges of *JunB*^*∆ep*^*S100a9*^*−/−*^ mice. μCT images are representative of at least 3 mice of each group. **b** SA CFUs in the skin of the digits of all 4 mutant mice. **c** Representative IF images of SA (red) and K5 (green) in the digits of *JunB*^*∆ep*^ and *JunB*^*∆ep*^*S100a9*^*−/−*^ mice. Nuclei are stained with DAPI (blue). Scale bar, 200μm. The white arrow points to SA penetration. **d** Percentage of CD45^+^ neutrophils (CD11b^+^, Ly6C^+^, Ly6G^+^) in the skin surrounding the digits of all mutant mice, analyzed by flow cytometry. **e** Protein levels of MPO, NE, A8, A9 and CP in skin lysates in the digits of control (n = 4), *S100a9*^*−/−*^ (n = 3 < 4)*, JunB*^*∆ep*^ (n = 9) and *JunB*^*∆ep*^*S100a9*^*−/−*^ (n = 6) mice normalized ([C]/[Cmax]) by row. The maximum absolute value ([Cmax]) in ng/ml is MPO: 40.33, NE: 25.25, A8: 9.72, A9: 3.44, CP:10.25. **f** Representative IF images of A8 (red) and K5 (green) in the skin surrounding the digits of *JunB*^*∆ep*^ and *JunB*^*∆ep*^*S100a9*^*−/−*^ mice. Nuclei are stained with DAPI (blue). The white arrow points to A8-positive epidermal cells. A white line indicates the limits of epidermis. Scale bar, 200μm. **g** Representative IF images of A8-positive cells (green) and Ly6G-positive neutrophils (red) in *JunB*^*∆ep*^ and *JunB*^*∆ep*^*S100a9*^*−/−*^ digits. Nuclei are stained with DAPI (blue). The white arrows point to A8- and Ly6G-stained cells. Scale bar, 200μm. Dot plots represent mean ± SEM. *p ≤ 0.05, **p ≤ 0.01, ***p ≤ 0.001, ****p ≤ 0.0001. Heat map represents [C]/[Cmax] means, scaled by row, *p ≤ 0.05, **p ≤ 0.01, ***p ≤ 0.001 compared to control mice, ^•^*p* ≤ 0.05 ^••^*p* ≤ 0.01, ^•••^*p* ≤ 0.001 compared to S100a9^−/−^ mice and ^*#*^*p* ≤ 0.05, ^##^*p* ≤ 0.01, ^###^*p* ≤ 0.001 compared to *JunB*^*∆ep*^ mice. One-way ANOVA with Fishers’ LSD test was used for statistical grouped analysis and unpaired 2-tailed Student’s t-test with Welch’s correction was applied to compare statistical difference between 2 groups (gray stars).

Unexpectedly, *JunB*^*∆ep*^*S100a9*^*−/−*^ mice developed prominent swelling of the digits with increased epidermal thickness (Fig. 5a, Fig. S5a). This was not observed in *JunB*^*∆ep*^ mice lacking A9 in keratinocytes or in neutrophils. Swollen digits were associated with local bone destruction in the distal phalanges as documented by μCT (Fig. 5a) and histology of digit sections with detectable TRAP^+^ bone resorbing osteoclasts (Fig. S5b, c). The skin of inflamed digits had significantly increased SA colonization (Fig. 5b) and the bacteria penetrated the skin of *JunB*^*∆ep*^*S100a9*^*−/−*^ digits (Fig. 5c). Bacteria sampled from the digits of *JunB*^*∆ep*^*S100a9*^*−/−*^ mice were predominantly *Staphylococci* and not different from those sampled from the snout of *JunB*^*∆ep*^ mice (Fig. S5d, Table S1). The skin isolated from *JunB*^*∆ep*^*S100a9*^*−/−*^ inflamed digits displayed a 2-fold increase in neutrophilic infiltrates (Fig. 5d) and an even more prominent increase in MPO and NE (Fig. 5e), when compared to digits from *JunB*^*∆ep*^ mice. A8 dimers and *A8* mRNA were also elevated in the skin isolated from the digits of *JunB*^*∆ep*^*S100a9*^*−/−*^ mice (Fig. 5e, Fig. S5d) and colonizing distal phalanges (Fig. S5e). A8 protein expression appeared more prominent in the thickened epidermis than in infiltrating neutrophils of *JunB*^*∆ep*^*S100a9*^*−/−*^ digits (Fig. 5f, g). Neutrophils isolated from the skin of *JunB*^*∆ep*^*S100a9*^*−/−*^ and *JunB*^*∆ep*^ digits had comparable *A8* mRNA expression (Fig. S5f). This indicates that keratinocytes and to a lesser extent neutrophils contribute to the increase in *A8* expression and A8 dimers in *JunB*^*∆ep*^*S100a9*^*−/−*^ digit skin. Interestingly, although *A8* and *A9* mRNA were increased in digit skin isolated from *JunB*^*∆ep*^ mice (Fig. S5f), A8 and A9 dimers, as well as CP were either not or only moderately increased in digit skin isolated from *JunB*^*∆ep*^ mice, when compared to controls (Fig. 5e), while all three complexes are elevated in snout skin extracts even before the lesions appear (Fig. 1d, Fig. S1g, h). This suggests that the local increase in A8 dimers is likely responsible for the digit phenotype in *JunB*^*∆ep*^*S100a9*^*−/−*^ mice. Importantly, the phenotype of *JunB*^*∆ep*^*S100a9*^*−/−*^ mice was largely recapitulated in *JunB*^*∆ep*^*S100a9*^*∆ep*^ mice reconstituted with BM cells from *A9*^*∆n*^ mice (Fig. S5h). Facial skin disease was less severe in *A9*^*∆n*^::*JunB*^*∆ep*^*S100a9*^*∆ep*^ mice compared to *JunB*^*∆ep*^ mice that received BM with A9-proficient neutrophils (control*:: JunB*^*∆ep*^*S100a9*^*∆ep*^) with no visible macroscopic skin lesions (Fig. S5i), no SA growth (Fig. S5j) and reduced neutrophilic infiltration in the snout (Fig. S5k). Neutrophils were also significantly increased in BM of *A9*^*∆n*^::*JunB*^*∆ep*^*S100a9*^*∆ep*^ mice (Fig. S5l), whereas other systemic manifestations, such as body weight loss and splenomegaly were marginally worsened in *A9*^*∆n*^::*JunB*^*∆ep*^*S100a9*^*∆ep*^ BM chimeras (Fig. S5m). Importantly, *A9*^*∆n*^::*JunB*^*∆ep*^*S100a9*^*∆ep*^ mice developed swollen digits (Fig. S5i) similar to those observed in *JunB*^*∆ep*^*S100a9*^*−/−*^ mice with SA colonization (Fig. S5j) and increased neutrophils (Fig. S5k). Collectively, A8 dimers appear to exert immune-modulatory functions in *JunB*^*∆ep*^ mice.

### Genetic inactivation of *A8* in epidermal cells improves skin and systemic disease in *JunB*^*∆ep*^ mice

**Fig. 6:**
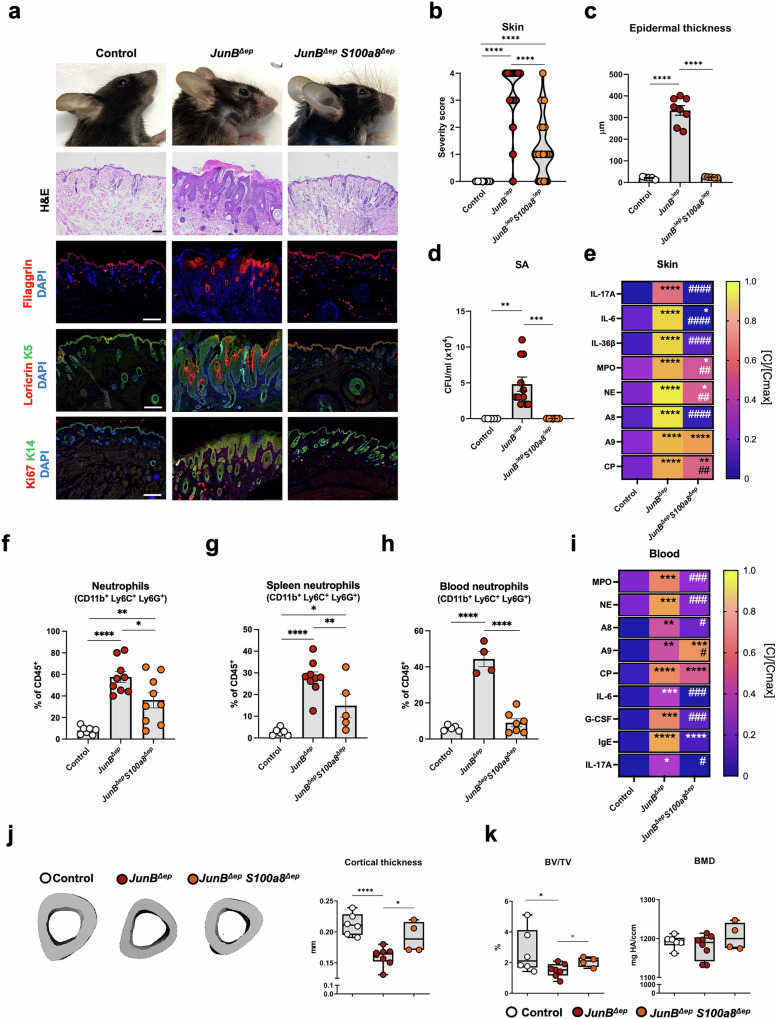
Loss of A8 in epidermal cells improves skin and systemic disease in *JunB*^*∆ep*^ mice. **a** Representative pictures of control, *JunB*^*∆ep*^ and *JunB*^*∆ep*^*S100a8*^*∆ep*^ mice, H&E staining of snout skin sections in indicated mice and IF of filaggrin, loricrin and Ki67 (red) co-stained with K5 or K14 (green), as indicated. Nuclei are stained with DAPI. Black scale bar, 100μm. White scale bars, 200μm. **b** Skin disease severity scoring from 0 (no lesions) to 4 (severe lesions in the face and ventral skin) of control, *JunB*^*∆ep*^ and *JunB*^*∆ep*^*S100a8*^*∆ep*^ mice. **c** Epidermal thickness (µm) measured on facial skin sections of mutant mice. Each dot represents the mean of 4 measurements for a single mouse. **d** SA CFUs in the skin of the indicated mice. **e** Protein levels of IL-17A, IL-6, IL-36β, MPO, NE, A8, A9 and CP in skin lysates from control (n = 5 < 6), *JunB*^*∆ep*^ (n = 5 < 7) and *JunB*^*∆ep*^ *S100a8*^*∆ep*^ (n = 8 < 9) mice normalized ([C]/[Cmax]) by row. The maximum absolute value ([Cmax]) in ng/ml is IL-17A: 2.72, IL-6: 1.79, IL-36β: 1.2, MPO: 22.04, NE: 2.36, A8: 5.63, A9: 4.46, CP: 9.63. **f** Percentage of CD45^+^ skin neutrophils (CD11b^+^, Ly6C^+,^ Ly6G^+^) in the indicated mice. **g** Flow cytometric analysis of splenic neutrophils (CD11b^+^, Ly6C^+^ and Ly6G^+^) in indicated mice, shown as percentage (%) of CD45^+^ cells in control, *JunB*^*∆ep*^ and *JunB*^*∆ep*^ *S100a8*^*∆ep*^ mice. **h** Blood granulocyte counts in control, *JunB*^*∆ep*^ and *JunB*^*∆ep*^*S100a8*^*∆ep*^ mice. **i** Protein levels of circulating MPO, NE, A8, A9, CP, IgE and IL-17A in the serum of control (n = 4 < 7), *JunB*^*∆ep*^ (n = 3 < 9) and *JunB*^*∆ep*^ *S100a8*^*∆ep*^ (n = 5 < 7) mice normalized ([C]/[Cmax]) by row. The maximum absolute value ^*[*^)([Cmax]) in ng/ml is MPO: 30.68, NE: 6.35, A8: 0.53, A9: 0.73, CP: 37.95, IgE: 5499.17, IL-17A: 0.74. **j** Representative μCT images of the tibiae (left) and quantification of the cortical thickness of the tibiae (right). **k** Quantification of BV/TV and BMD of the tibiae in the indicated groups. Dot plots represent mean ± SEM. *p ≤ 0.05, **p ≤ 0.01, ***p ≤ 0.001, ****p ≤ 0.0001. Heat maps represent [C]/[Cmax] means, scaled by row, *p ≤ 0.05, **p ≤ 0.01, ***p ≤ 0.001, ****p ≤ 0.0001 compared to control mice and ^#^*p* ≤ 0.05, ^##^*p* ≤ 0.01, ^###^*p* ≤ 0.001, ^####^*p* ≤ 0.0001 compared to *JunB*^*∆ep*^ mice. One-way ANOVA with Fishers’ LSD test was used for statistical grouped analysis, and unpaired 2-tailed Student’s t-test with Welch’s correction was applied to compare statistical difference between 2 groups (gray stars).

*JunB*^*∆ep*^*S100a8*^*∆ep*^ mice were next generated to define the role of keratinocyte-derived A8 in chronic skin inflammation. In sharp contrast to epithelial-specific *A9* inactivation, a notable improvement of the disease was observed with less skin lesions (Fig. 6a, b), a marked reduction of epidermal thickening (Fig. 6a, c, Fig. S6a), reduced Ki67^+^, K5^+^ and K14^+^ proliferative keratinocytes, and restored filaggrin and loricrin expression patterns suggesting an improved epidermal barrier (Fig. 6a, Fig. S6a). Consistently, spontaneous SA colonization was reduced in the snout of *JunB*^*∆ep*^*S100a8*^*∆ep*^ mice, when compared to *JunB*^*∆ep*^ littermates (Fig. 6d). Reduced IL-17A, IL-6, IL-36β, MPO and NE (Fig. 6e) were observed in the skin of *JunB*^*∆ep*^*S100a8*^*∆ep*^ mice, along with decreased CD45^+^ immune cells and neutrophils (Fig. 6f, Fig. S6b). Importantly, A8 dimers were greatly reduced in *JunB*^*∆ep*^*S100a8*^*∆ep*^ snout extracts, when compared to *JunB*^*∆ep*^ mutants, reaching the levels measured in controls, while the decrease in CP was less prominent and A9 dimers unchanged (Fig. 6e).

Keratinocyte-specific inactivation of *A8* appeared beneficial systemically, as body weight loss and splenomegaly were largely alleviated in *JunB*^*∆ep*^*S100a8*^*∆ep*^ mice (Fig. S6c). Splenic neutrophils, BM CD45^+^ immune cells and blood granulocytes and neutrophils were also decreased in *JunB*^*∆ep*^*S100a8*^*∆ep*^ mice, when compared to *JunB*^*∆ep*^ littermates (Fig. 6g, h, Fig. S6d, e), while BM neutrophils and blood lymphocytes were unchanged (Fig. S6d, f). *JunB*^*∆ep*^*S100a8*^*∆ep*^ mice had reduced circulating MPO, NE, A8 dimers and CP, similar to what was observed in skin extracts from the snout, but higher A9 dimers (Fig. 6i). IgE, IL-17A, IL-6 and G-CSF were decreased compared to *JunB*^*∆ep*^ mice and similar to control levels (Fig. 6i). Consistent with decreased circulating IL-17A, bone loss was ameliorated in *JunB*^*∆ep*^*S100a8*^*∆ep*^ compared to *JunB*^*∆ep*^ mice with increased cortical thickness and BV/TV, while BMD remained comparable to controls (Fig. 6j, k). These data indicate that genetic inactivation of *A8* in epidermal cells improves the skin and systemic manifestations of experimental chronic skin inflammation, in stark contrast to epidermal *A9* inactivation.

### Loss of A8 in epidermal cells promotes SA colonization and bone destruction in *JunB*^*∆ep*^ digits

**Fig. 7:**
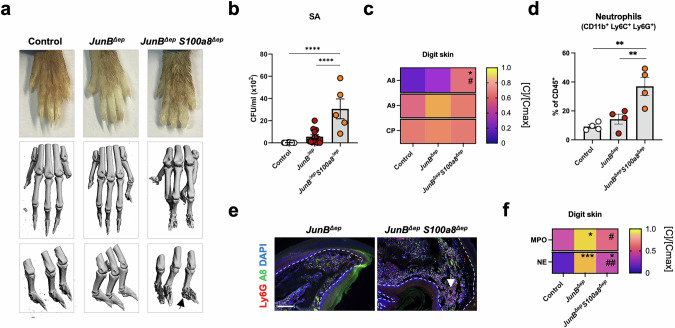
Genetic inactivation of *A8* in epidermal cells promotes SA colonization and bone destruction in *JunB*^*∆ep*^ digits. **a** Representative images of hind paws of control, *JunB*^*∆ep*^ and *JunB*^*∆ep*^*S100a8*^*∆ep*^ mice (top panel) and μCT images of the digits (middle and bottom panels). The black arrow indicates bone destruction in the distal phalanges of *JunB*^*∆ep*^*S100a8*^*∆ep*^ mice. **b** SA CFUs in the skin of the digits of all 3 group of mice. **c** Protein levels of A8, A9 and CP in the skin of control (n = 3 < 4), *JunB*^*∆ep*^ (n = 5 < 8) and *JunB*^*∆ep*^*S100a8*^*∆ep*^ (n = 5) digits normalized ([C]/[Cmax]) by row. The maximum absolute value ([Cmax]) in ng/ml is A8: 4.36, A9: 3.22, CP: 5.57. **d** Percentage of CD45^+^ neutrophils (CD11b^+^, Ly6C^+^, Ly6G^+^) in the skin of control, *JunB*^*∆ep*^ and *JunB*^*∆ep*^*S100a8*^*∆ep*^ digits. **e** IF of A8- (green) and Ly6G-positive cells (red) in *JunB*^*∆ep*^ and *JunB*^*∆ep*^*S100a8*^*∆ep*^ digits. Nuclei are stained with DAPI. The white arrow indicates A8- and Ly6G-double stained neutrophils. White lines indicate the limits of epidermis. Scale bars, 200μm. **f** Protein levels of MPO and NE in the skin of control (n = 3), *JunB*^*∆ep*^ (n = 5) and *JunB*^*∆ep*^*S100a8*^*∆ep*^ (n = 5) digits normalized ([C]/[Cmax]) by row. The maximum absolute value ([Cmax]) in ng/ml is MPO: 3.13, NE: 1.70. Dot plots represent mean ± SEM. *p ≤ 0.05, **p ≤ 0.01, ***p ≤ 0.001, ****p ≤ 0.0001. Heat maps represent [C]/[Cmax] means, scaled by row, *p ≤ 0.05, **p ≤ 0.01, ***p ≤ 0.001, compared to control mice and ^#^*p* ≤ 0.05, ^##^*p* ≤ 0.01 compared to *JunB*^*∆ep*^ mice. One-way ANOVA with Fishers’ LSD test was used for statistical grouped analysis.

Although the digits of *JunB*^*∆ep*^*S100a8*^*∆ep*^ mice were not overtly swollen, bone destruction in the distal phalanges and SA colonization in the skin of the digits were observed (Fig. 7a, b), reminiscent of *JunB*^*∆ep*^*S100a9*^*−/−*^ mice (Fig. 5a, b). No deeper bacterial colonization was observed reaching the distal phalanges (Fig. S6g). Interestingly, A8 dimers were significantly higher in *JunB*^*∆ep*^*S100a8*^*∆ep*^ digit skin extracts (Fig. 7c), similar to *JunB*^*∆ep*^*S100a9*^*−/−*^ digits (Fig. 5e). A9 dimers and CP were also detectable in the skin of *JunB*^*∆ep*^*S100a8*^*∆ep*^ digits, although not different from *JunB*^*∆ep*^ or control mice (Fig. 7c). Increased skin-infiltrating neutrophils was measured in *JunB*^*∆ep*^*S100a8*^*∆ep*^ digits by flow cytometry (Fig. 7d) and IF staining revealed Ly6G- and A8-double positive neutrophils in the dermis of *JunB*^*∆ep*^*S100a8*^*∆ep*^ digits (Fig. 7e), although MPO and NE levels were reduced in total digit skin extracts (Fig. 7f). These data establish a novel cell- and site-specific function of A8 modulating local inflammation and suggest that A8 dimers are pro-inflammatory and contribute to SA invasion and bone destruction in chronic skin inflammation.

## Discussion

Inflammatory skin diseases (ISDs) are chronic and severe diseases with a pathogenesis that remains incompletely understood. The chronicity of skin inflammation suggests fundamental dysregulation of ISD-associated skin proteins. The S100A8 (A8) and S100A9 (A9) alarmins have been implicated in skin inflammation with yet unclear functions. In this study, we define a protective role of epidermal A9 and a pro-inflammatory function of epidermal A8, as well as divergent functions of A9 expression in keratinocytes and neutrophils in modulating experimental skin inflammation and its extra-cutaneous manifestations. Loss of A9 in epidermal cells enhanced, while loss of neutrophil-derived A9 and *A9* knock-out improved skin inflammation in GEMMs, similar to what was observed in *A9* knock-out and epidermal A9-deficient psoriasis (Ps)-like mice [26, 27]. In contrast, *A8* inactivation in epidermal cells improved chronic skin and systemic inflammation.

The worsening of skin disease observed in the absence of epidermal A9 correlated with a more pronounced infiltration of A9-positive neutrophils, a process that is similar to what was observed in Ps-like mice [27]. It is likely that neutrophils are at least partially responsible for the worsened skin disease, since *JunB*^*∆ep*^ mice reconstituted with neutrophil A9-deficient BM displayed ameliorated skin inflammation with reduced IL-6 levels, consistent with previous findings reporting that IL-6 is a downstream target of JunB in keratinocytes [25]. Improved epidermal barrier alterations and reduced epidermal thickening, similar to Ps-like mice with transplanted *A9* knock-out BM [26] was observed. Importantly, systemic inflammation was not abolished in BM chimeras, suggesting that other factors such as A8, are required to develop the systemic disease. *JunB*^*∆ep*^*S100a9*^*−/− *^mice exhibited an improvement of skin inflammation with reduced A8 in the snout and ventral skin. Thus, the pro-inflammatory function of A9-expressing neutrophils is dominant over the anti-inflammatory role of A9-expressing keratinocytes modulating the skin manifestations of experimental ISD. The lack of A9 in neutrophils reduced local A8 and led to an increase in Calprotectin (CP) levels in the skin of the mutants, suggesting that epidermal CP may also play a “protecting/calming” function as described in Inflammatory Bowel Disease [31, 34, 40]. Furthermore, *A9* knock-out in *JunB*^*∆ep*^ mice, which leads to systemic loss of CP [26, 34, 37, 40–42], aggravated systemic neutrophilic inflammation and amplified bone loss, the latter likely due to increased circulating IL-17A as previously described in *JunB*^*∆ep*^ mice [23]. These data suggest that A8 and A9 dimers along with CP hetero-complexes play divergent functions locally or systemically downstream of JunB. Furthermore, complete A9 deficiency reduced A8 dimers in snout and ventral skin, but increased A8 protein levels in the skin of the digits, suggesting that A8 and A9 dimers exert site-specific functions within the skin. Antagonistic functions of A8 and A9 dimers have been described depending on the quaternary structure, leading to differential binding abilities to TLR4, RAGE and CD69. A8/A9 heterodimers can mediate inflammation by binding to RAGE and TLR4, whereas calcium-induced A8/A9 tetramer formation prevents this interaction restricting inflammation and avoiding systemic inflammation [30, 36]. Binding of CP to TLR4 or CD69 receptors is responsible for mediating its opposing functions in monocytes [34, 36], where A8/A9 heterodimer binds to TLR4 promoting pro-inflammatory functions, whereas the A8/A9 tetramer binds to CD69 to dampen monocyte dynamics including adhesion and migration. This may help explain why certain skin parts are more prone to develop inflammation and macroscopic lesions than others [3], a fact that is also attributed to the skin diverse composition, distinct pH, temperature, sebum content and hair follicle patterns [43, 44].

Complete *A9* inactivation can increase in mice the susceptibility to bacterial infections, such as SA-induced pneumonia [45]. The absence of A9 in *JunB*^*∆ep*^ mice led to invasive SA infection in the digits, which reached deeper skin layers. SA colonization and the intensity of itching largely correlates with AD severity [46, 47]. Therefore, epidermal penetration by SA may contribute to the massive production of IgE observed in *A9* knock-out *JunB*^*∆ep*^ mice. SA skin exposure and high IgE leads to a scratch behavior and mechanical injury in mice with epidermal-barrier disruption, highlighting the significance of physical stress on the observed phenotypes [48–50]. Compromised epidermal barrier in the digits of *JunB*^*∆ep*^ mice in the absence of A9, may further contribute to SA penetration into deeper layers of the skin. Invasive SA infections and life-threatening complications can occur in poorly managed AD, including bone infection, known as osteomyelitis which leads to bone destruction particularly in the digits [8, 9, 51–53]. Importantly, SA colonization in the digits of *A9* knock-out *JunB*^*∆ep*^ mice was associated with destruction of distal phalanges, likely due to increased osteoclast numbers, whereas joints were not affected differing from the observations in PS-like disease. This suggests an involvement of distinct pathways in PS- and AD-like disease, likely dependent on different cytokines. The causal relationship between the observed SA colonization and the bone damage in distal phalanges remains to be investigated, although the increase in pro-inflammatory A8 in the digits could provide a first mechanistic hint.

This study is the first to investigate epidermal A8 function in vivo using a novel GEMM with epidermal loss of A8. Complete genetic inactivation of *A8* was reported to be embryonic lethal [54, 55], although one study described viable *A8* knock-out mice with enhanced Imiquimod-induced epidermal thickening and arthritis [56]. Investigating the role of A8 in SA infection and bone destruction may provide valuable insights for future research on ISDs and musculoskeletal diseases.

A correlation between diminished levels of A8 and the amelioration of lesional skin following AD treatment was reported [57], suggesting a pro-inflammatory function for A8. In light of our findings, topical application on lesional skin or subcutaneous administration of drugs that inhibit A8 might be worth evaluating in future studies. The divergent roles of A8 and A9 dimers are likely attributed to their distinct receptor interactions [36]. However, the cell-type-specific receptor interaction profiles of A8- or A9-containing complexes in ISD remain unexplored. Future studies are also needed to uncover the downstream signaling pathways through which A8, A9 and CP unleash anti- or pro-inflammatory programs in AD.

While GEMMs do not fully recapitulate human diseases and results obtained in mice may not directly translate to clinical settings due to species-specific differences in immune and inflammatory responses, GEMMs are a powerful tool for investigating the cell-type-specific effects and functions of genes in the whole organism.

In conclusion, our study shows for the first time cell- and site-specific effects of *A8* or *A9* genetic inactivation in the skin, demonstrating divergent actions of A8 and A9 in chronic skin and systemic inflammation with musculoskeletal consequences. The absence of A8 in lesional mouse skin reduces inflammation, suggesting a potential for further investigations into therapeutic approaches to restore epidermal homeostasis and prevent systemic complications in AD. Future studies may determine cell-specific functions of A8, A9 and CP on other clinically relevant issues, such as the relationship between AD and primary cutaneous T-cell lymphomas, which present important diagnostic challenges.

## Supplementary information

Supplemental Material

Figure S1

Figure S2

Figure S3

Figure S4

Figure S5

Figure S6

## Data Availability

The authors confirm that the data supporting the findings of this study are available within the article and/or the Supplementary Materials. Further information and requests for resources should be directed to the lead contact, Erwin F. Wagner (erwin.wagner@meduniwien.ac.at).

## References

[1] Hay RJ, Johns NE, Williams HC, Bolliger IW, Dellavalle RP, Margolis DJ, et al. The global burden of skin disease in 2010: an analysis of the prevalence and impact of skin conditions. J Invest Dermatol. 2014;134:1527–34. 24166134 10.1038/jid.2013.446

[2] Karimkhani C, Dellavalle RP, Coffeng LE, Flohr C, Hay RJ, Langan SM, et al. Global skin disease morbidity and mortality: an update from the global burden of disease study 2013. JAMA Dermatol. 2017;153:406–12. 28249066 10.1001/jamadermatol.2016.5538 PMC5817488

[3] Langan SM, Irvine AD, Weidinger S. Atopic dermatitis. Lancet. 2020;396:345–60. 32738956 10.1016/S0140-6736(20)31286-1

[4] Weidinger S, Beck LA, Bieber T, Kabashima K, Irvine AD. Atopic dermatitis. Nat Rev Dis Primers. 2018;4:1. 29930242 10.1038/s41572-018-0001-z

[5] Ober C, Yao TC. The genetics of asthma and allergic disease: a 21st century perspective. Immunol Rev. 2011;242:10–30. 21682736 10.1111/j.1600-065X.2011.01029.x PMC3151648

[6] Kobayashi T, Glatz M, Horiuchi K, Kawasaki H, Akiyama H, Kaplan DH, et al. Dysbiosis and *Staphylococcus aureus* colonization drives inflammation in atopic dermatitis. Immunity. 2015;42:756–66. 25902485 10.1016/j.immuni.2015.03.014 PMC4407815

[7] Geoghegan JA, Irvine AD, Foster TJ. *Staphylococcus aureus* and atopic dermatitis: a complex and evolving relationship. Trends Microbiol. 2018;26:484–97. 29233606 10.1016/j.tim.2017.11.008

[8] Masters EA, Ricciardi BF, Bentley KLM, Moriarty TF, Schwarz EM, Muthukrishnan G. Skeletal infections: microbial pathogenesis, immunity and clinical management. Nat Rev Microbiol. 2022;20:385–400. 35169289 10.1038/s41579-022-00686-0 PMC8852989

[9] Masuka JT, Troisi K, Mkhize Z. Osteomyelitis complicating secondarily infected atopic eczema: two case reports and a narrative literature review. BMC Dermatol. 2020;20:2. 32008574 10.1186/s12895-019-0098-0 PMC6996158

[10] Chopra R, Vakharia PP, Sacotte R, Silverberg JI. Efficacy of bleach baths in reducing severity of atopic dermatitis: A systematic review and meta-analysis. Ann Allergy Asthma Immunol. 2017;119:435–40. 29150071 10.1016/j.anai.2017.08.289 PMC5726436

[11] Sawada Y, Tong Y, Barangi M, Hata T, Williams MR, Nakatsuji T, et al. Dilute bleach baths used for treatment of atopic dermatitis are not antimicrobial in vitro. J Allergy Clin Immunol. 2019;143:1946–8. 30677478 10.1016/j.jaci.2019.01.009 PMC7183041

[12] Nakatsuji T, Hata TR, Tong Y, Cheng JY, Shafiq F, Butcher AM, et al. Development of a human skin commensal microbe for bacteriotherapy of atopic dermatitis and use in a phase 1 randomized clinical trial. Nat Med. 2021;27:700–9. 33619370 10.1038/s41591-021-01256-2 PMC8052297

[13] Werfel T, Allam JP, Biedermann T, Eyerich K, Gilles S, Guttman-Yassky E, et al. Cellular and molecular immunologic mechanisms in patients with atopic dermatitis. J Allergy Clin Immunol. 2016;138:336–49. 27497276 10.1016/j.jaci.2016.06.010

[14] Uluckan O, Wagner EF. Chronic systemic inflammation originating from epithelial tissues. FEBS J. 2017;284:505–16. 27650997 10.1111/febs.13904

[15] Garg NK, Silverberg JI. Eczema is associated with osteoporosis and fractures in adults: a US population-based study. J Allergy Clin Immunol. 2015;135:1085–7.e2. 25512080 10.1016/j.jaci.2014.10.043

[16] Shaheen MS, Silverberg JI. Atopic dermatitis is associated with osteoporosis and osteopenia in older adults. J Am Acad Dermatol. 2019;80:550–1. 29800580 10.1016/j.jaad.2018.05.026

[17] Moosbrugger-Martinz V, Schmuth M, Dubrac S. A mouse model for atopic dermatitis using topical application of vitamin D3 or of its analog MC903. Methods Mol Biol. 2017;1559:91–106. 28063040 10.1007/978-1-4939-6786-5_8

[18] Nakatsuji T, Brinton SL, Cavagnero KJ, O’Neill AM, Chen Y, Dokoshi T, et al. Competition between skin antimicrobial peptides and commensal bacteria in type 2 inflammation enables survival of *S. aureus*. Cell Rep. 2023;42:112494. 37167061 10.1016/j.celrep.2023.112494 PMC10303920

[19] Zenz R, Eferl R, Kenner L, Florin L, Hummerich L, Mehic D, et al. Psoriasis-like skin disease and arthritis caused by inducible epidermal deletion of Jun proteins. Nature. 2005;437:369–75. 16163348 10.1038/nature03963

[20] Zenz R, Eferl R, Scheinecker C, Redlich K, Smolen J, Schonthaler HB, et al. Activator protein 1 (Fos/Jun) functions in inflammatory bone and skin disease. Arthritis Res Ther. 2008;10:201. 18226189 10.1186/ar2338 PMC2374460

[21] Uluckan O, Jimenez M, Roediger B, Schnabl J, Diez-Cordova LT, Troule K, et al. Cutaneous immune cell-microbiota interactions are controlled by epidermal JunB/AP-1. Cell Rep. 2019;29:844–59.e3. 31644908 10.1016/j.celrep.2019.09.042 PMC6856727

[22] Sukseree S, Bakiri L, Irigoyen MP, Uluckan O, Petzelbauer P, Wagner EF. Sequestosome 1/p62 enhances chronic skin inflammation. J Allergy Clin Immunol. 2021;147:2386–93.e4. 10.1016/j.jaci.2021.02.028 33675820

[23] Uluckan O, Jimenez M, Karbach S, Jeschke A, Grana O, Keller J, et al. Chronic skin inflammation leads to bone loss by IL-17-mediated inhibition of Wnt signaling in osteoblasts. Sci Transl Med. 2016;8:330ra37. 27089206 10.1126/scitranslmed.aad8996

[24] Meixner A, Zenz R, Schonthaler HB, Kenner L, Scheuch H, Penninger JM, et al. Epidermal JunB represses G-CSF transcription and affects haematopoiesis and bone formation. Nat Cell Biol. 2008;10:1003–11. 18641637 10.1038/ncb1761

[25] Pflegerl P, Vesely P, Hantusch B, Schlederer M, Zenz R, Janig E, et al. Epidermal loss of JunB leads to a SLE phenotype due to hyper IL-6 signaling. Proc Natl Acad Sci USA. 2009;106:20423–8. 19918056 10.1073/pnas.0910371106 PMC2787143

[26] Schonthaler HB, Guinea-Viniegra J, Wculek SK, Ruppen I, Ximenez-Embun P, Guio-Carrion A, et al. S100A8-S100A9 protein complex mediates psoriasis by regulating the expression of complement factor C3. Immunity. 2013;39:1171–81. 24332034 10.1016/j.immuni.2013.11.011

[27] Mellor LF, Gago-Lopez N, Bakiri L, Schmidt FN, Busse B, Rauber S, et al. Keratinocyte-derived S100A9 modulates neutrophil infiltration and affects psoriasis-like skin and joint disease. Ann Rheum Dis. 2022;81:1400–8. 35788494 10.1136/annrheumdis-2022-222229 PMC9484400

[28] Manils J, Webb LV, Howes A, Janzen J, Boeing S, Bowcock AM, et al. CARD14(E138A) signalling in keratinocytes induces TNF-dependent skin and systemic inflammation. Elife. 2020;9:e56720. 10.7554/eLife.56720 PMC7351492 32597759

[29] Petersen B, Wolf M, Austermann J, van Lent P, Foell D, Ahlmann M, et al. The alarmin Mrp8/14 as regulator of the adaptive immune response during allergic contact dermatitis. EMBO J. 2013;32:100–11. 23188082 10.1038/emboj.2012.309 PMC3545303

[30] Vogl T, Stratis A, Wixler V, Voller T, Thurainayagam S, Jorch SK, et al. Autoinhibitory regulation of S100A8/S100A9 alarmin activity locally restricts sterile inflammation. J Clin Invest. 2018;128:1852–66. 29611822 10.1172/JCI89867 PMC5919817

[31] von Wulffen M, Luehrmann V, Robeck S, Russo A, Fischer-Riepe L, van den Bosch M, et al. S100A8/A9-alarmin promotes local myeloid-derived suppressor cell activation restricting severe autoimmune arthritis. Cell Rep. 2023;42:113006. 37610870 10.1016/j.celrep.2023.113006

[32] Benhadou F, Glitzner E, Brisebarre A, Swedlund B, Song Y, Dubois C, et al. Epidermal autonomous VEGFA/Flt1/Nrp1 functions mediate psoriasis-like disease. Sci Adv. 2020;6:eaax5849. 31934626 10.1126/sciadv.aax5849 PMC6949033

[33] Silva de Melo BM, Veras FP, Zwicky P, Lima D, Ingelfinger F, Martins TV, et al. S100A9 drives the chronification of psoriasiform inflammation by inducing IL-23/Type 3 immunity. J Invest Dermatol. 2023;143:1678–88.e8. 36921684 10.1016/j.jid.2023.02.026

[34] Russo A, Schurmann H, Brandt M, Scholz K, Matos ALL, Grill D, et al. Alarming and Calming: Opposing Roles of S100A8/S100A9 Dimers and Tetramers on Monocytes. Adv Sci. 2022;9:e2201505. 10.1002/advs.202201505 PMC9798971 36310133

[35] Defrene J, Berrazouane S, Esparza N, Page N, Cote MF, Gobeil S, et al. Deletion of S100a8 and S100a9 enhances skin hyperplasia and promotes the Th17 response in imiquimod-induced psoriasis. J Immunol. 2021;206:505–14. 33361205 10.4049/jimmunol.2000087

[36] Wang S, Song R, Wang Z, Jing Z, Wang S, Ma J. S100A8/A9 in Inflammation. Front Immunol. 2018;9:1298. 29942307 10.3389/fimmu.2018.01298 PMC6004386

[37] Jukic A, Bakiri L, Wagner EF, Tilg H, Adolph TE. Calprotectin: from biomarker to biological function. Gut. 2021;70:1978–88. 34145045 10.1136/gutjnl-2021-324855 PMC8458070

[38] Rojahn TB, Vorstandlechner V, Krausgruber T, Bauer WM, Alkon N, Bangert C, et al. Single-cell transcriptomics combined with interstitial fluid proteomics defines cell type-specific immune regulation in atopic dermatitis. J Allergy Clin Immunol. 2020;146:1056–69. 32344053 10.1016/j.jaci.2020.03.041

[39] Passegue E, Wagner EF, Weissman IL. JunB deficiency leads to a myeloproliferative disorder arising from hematopoietic stem cells. Cell. 2004;119:431–43. 15507213 10.1016/j.cell.2004.10.010

[40] Steinbakk M, Naess-Andresen CF, Lingaas E, Dale I, Brandtzaeg P, Fagerhol MK. Antimicrobial actions of calcium binding leucocyte L1 protein, calprotectin. Lancet. 1990;336:763–5. 1976144 10.1016/0140-6736(90)93237-j

[41] Austermann J, Spiekermann C, Roth J. S100 proteins in rheumatic diseases. Nat Rev Rheumatol. 2018;14:528–41. 30076385 10.1038/s41584-018-0058-9

[42] Kehl-Fie TE, Zhang Y, Moore JL, Farrand AJ, Hood MI, Rathi S, et al. MntABC and MntH contribute to systemic *Staphylococcus aureus* infection by competing with calprotectin for nutrient manganese. Infect Immun. 2013;81:3395–405. 23817615 10.1128/IAI.00420-13 PMC3754211

[43] Chen YE, Fischbach MA, Belkaid Y. Skin microbiota-host interactions. Nature. 2018;553:427–36. 29364286 10.1038/nature25177 PMC6075667

[44] Belkaid Y, Segre JA. Dialogue between skin microbiota and immunity. Science. 2014;346:954–9. 25414304 10.1126/science.1260144

[45] De Filippo K, Neill DR, Mathies M, Bangert M, McNeill E, Kadioglu A, et al. A new protective role for S100A9 in regulation of neutrophil recruitment during invasive pneumococcal pneumonia. FASEB J. 2014;28:3600–8. 24776746 10.1096/fj.13-247460

[46] Stander S. Atopic dermatitis. N Engl J Med. 2021;384:1136–43. 33761208 10.1056/NEJMra2023911

[47] Gong JQ, Lin L, Lin T, Hao F, Zeng FQ, Bi ZG, et al. Skin colonization by *Staphylococcus aureus* in patients with eczema and atopic dermatitis and relevant combined topical therapy: a double-blind multicentre randomized controlled trial. Br J Dermatol. 2006;155:680–7. 16965415 10.1111/j.1365-2133.2006.07410.x

[48] Williams MR, Nakatsuji T, Gallo RL. *Staphylococcus aureus*: master manipulator of the skin. Cell Host Microbe. 2017;22:579–81. 29120738 10.1016/j.chom.2017.10.015 PMC5814126

[49] Deng L, Costa F, Blake KJ, Choi S, Chandrabalan A, Yousuf MS, et al. S. aureus drives itch and scratch-induced skin damage through a V8 protease-PAR1 axis. Cell. 2023;186:5375–93.e25. 37995657 10.1016/j.cell.2023.10.019 PMC10669764

[50] Kopp EB, Agaronyan K, Licona-Limon I, Nish SA, Medzhitov R. Modes of type 2 immune response initiation. Immunity. 2023;56:687–94. 37044059 10.1016/j.immuni.2023.03.015

[51] Cassat JE, Hammer ND, Campbell JP, Benson MA, Perrien DS, Mrak LN, et al. A secreted bacterial protease tailors the *Staphylococcus aureus* virulence repertoire to modulate bone remodeling during osteomyelitis. Cell Host Microbe. 2013;13:759–72. 23768499 10.1016/j.chom.2013.05.003 PMC3721972

[52] Patel D, Jahnke MN. Serious complications from Staphylococcal aureus in atopic dermatitis. Pediatr Dermatol. 2015;32:792–6. 26337792 10.1111/pde.12665

[53] Boiko S, Kaufman RA, Lucky AW. Osteomyelitis of the distal phalanges in three children with severe atopic dermatitis. Arch Dermatol. 1988;124:418–23. 3345091

[54] Baker JR, Jeffery R, May RD, Mathies M, Spencer-Dene B, Poulsom R, et al. Distinct roles for S100a8 in early embryo development and in the maternal deciduum. Dev Dyn. 2011;240:2194–203. 22016186 10.1002/dvdy.22709

[55] Passey RJ, Xu K, Hume DA, Geczy CL. S100A8: emerging functions and regulation. J Leukoc Biol. 1999;66:549–56. 10534107 10.1002/jlb.66.4.549

[56] Cesaro A, Defrene J, Lachhab A, Page N, Tardif MR, Al-Shami A, et al. Enhanced myelopoiesis and aggravated arthritis in S100a8-deficient mice. PLoS ONE. 2019;14:e0221528. 31437241 10.1371/journal.pone.0221528 PMC6705798

[57] Guttman-Yassky E, Bissonnette R, Ungar B, Suarez-Farinas M, Ardeleanu M, Esaki H, et al. Dupilumab progressively improves systemic and cutaneous abnormalities in patients with atopic dermatitis. J Allergy Clin Immunol. 2019;143:155–72. 30194992 10.1016/j.jaci.2018.08.022

