# Supplementary material for: Chronic skin and systemic inflammation modulated by S100A8 and S100A9 complexes
Source: Cell Death Differ. 2025 Apr 11;32(10):1833–44. doi: 10.1038/s41418-025-01504-9 (PMC12501315; doi:10.1038/s41418-025-01504-9)
Supplement: Supplementary file 1 — Supplemental Material [file 41418_2025_1504_MOESM1_ESM.docx]

**SUPPLEMENTARY MATERIALS**

**Supplementary Tables**

**Supplementary Figure legends**

**Supplementary Methods**

**Supplementary References**

**Table S1. 16S rRNA sequencing results.** Identified species with highest identity (%) were assigned to ASVs. Titles describing information below are highlighted in bold.

| **ASV#** | **Read** |
| --- | --- |
| ASV1 | GATGAACGCTGGCGGCGTGCCTAATACATGCAAGTCGAGCGAACGGACGAGAAGCTTGCTTCTCTGATGTTAGCGGCGGACGGGTGAGTAACACGTGGATAACCTACCTATAAGACTGGGATAACTTCGGGAAACCGGAGCTAATACCGGATAATATTTTGAACCGCATGGTTCAAAAGTGAAAGACGGTCTTGCTGTCACTTATAGATGGATCCGCGCTGCATTAGCTAGTTGGTAAGGTAACGGCTTACCAAGGCAACGATGCATAGCCGACCTGAGAGGGTGATCGGCCACACTGGAACTGAGACACGGTCCAG |
| ASV2 | GATGAACGCTGGCGGCGTGCCTAATACATGCAAGTCGAGCGAACAGATAAGGAGCTTGCTCCTTTGAAGTTAGCGGCGGACGGGTGAGTAACACGTGGGTAACCTACCTATAAGACTGGGATAACTTCGGGAAACCGGAGCTAATACCGGATAACATTTAGAACCGCATGGTTCTAAAGTGAAAGATGGTTTTGCTATCACTTATAGATGGACCCGCGCCGTATTAGCTAGTTGGTAAGGTAACGGCTTACCAAGGCGACGATACGTAGCCGACCTGAGAGGGTGATCGGCCACACTGGAACTGAGACACGGTCCAG |
| ASV3 | GATGAACGCTAGCGGCAGGCTTAACACATGCAAGTCGAGGGGCAGCGGGGAGGAAGCTTGCTTTCTCCGCCGGCGACCGGCGCACGGGTGAGTAACACGTATGCAACCTGCCCCTGTCAGGGGGACAACCCGCCGAAAGGCGGGCTAAACCCGCGTACATGCCACCGGGGCATCCCGGAGGCAGGAAAGGCTTCGGCCGGACAGGGATGGGCATGCGGCGCATTAGGCAGTAGGCGGGGTAACGGCCCACCTAACCGACGATGCGTAGGGGTTCTGAGAGGAAGGTCCCCCACACTGGTACTGAGACACGGACCAG |
| ASV5 | GATGAACGCTGGCGGCGTGCCTAATACATGCAAGTCGAGCGAACGGACGAGAAGCTTGCTTCTCTGATGTTAGCGGCGGACGGGTGAGTAACACGTGGACAACCTACCTATAAGACTGGGATAACTTCGGGAAACCGGAGCTAATACCGGATAATATTTTGAACCGCATGGTTCAAAAGTGAAAGACGGTCTTGCTGTCACTTATAGATGGATCCGCGCTGCATTAGCTAGTTGGTAAGGTAACGGCTTACCAAGGCAACGATGCATAGCCGACCTGAGAGGGTGATCGGCCACACTGGAACTGAGACACGGTCCAG |
| ASV9 | GACGAACGCTGGCGGCGTGCCTAATACATGCAAGTCGAGCGAGCTTGCCTAGATGATTTTAGTGCTTGCACTAAATGAAACTAGATACAAGCGAGCGGCGGACGGGTGAGTAACACGTGGGTAACCTGCCCAAGAGACTGGGATAACACCTGGAAACAGATGCTAATACCGGATAACAACACTAGACGCATGTCTAGAGTTTGAAAGATGGTTCTGCTATCACTCTTGGATGGACCTGCGGTGCATTAGCTAGTTGGTAAGGTAACGGCTTACCAAGGCAATGATGCATAGCCGAGTTGAGAGACTGATCGGCCACATTGGGACTGAGACACGGCCCAA |
| ASV10 | GATGAACGCTGGCGGCGTGCTTAACACATGCAAGTCGAACGGACTCATATTGAAACCTAGTGATTTATGAGTTAGTGGCGGACGGGTGAGTAACGCGTGGAAAACCTGCCGTATACTGGGGGATAACACTTAGAAATAGGTGCTAATACCGCATAAGCGCACAGCTTCGCATGAAGTGGTGTGAAAAACTCTGGTGGTATACGATGGTTCCGCGTCTGATTAGCTTGTTGGTGGGGTAATGGCTCACCAAGGCGACGATCAGTAGCCGGCCTGAGAGGGTGAACGGCCACATTGGGACTGAGACACGGCCCAA |
| ASV20 | GATGAACGCTAGCGGCAGGCTTAACACATGCAAGTCGAGGGGCAGCATGGGGAGTAGCAATACTCTCTGATGGCGACCGGCGCAAGGGTGCGTAACGCGTGAGCAACTTGCCCTCATCAGGGGAATAATCGCTGGAAACGGCGTCTAATGCCCCATGGTGATGTCCTCAGGCATCTGAGGTCATCTAAAGATCCGTCGGATGGGGATAGGCTCGCGTGACATTAGCTAGACGGCGGGGTAACGGCCCACCGTGGCGACGATGTCTAGGGGTTCTGAGAGGAAGGTCCCCCACACTGGAACTGAGACACGGTCCAG |
| ASV69 | GATGAACGCTGGCGGCGTGCCTAATACATGCAAGTCGAGCGAACGGACGAGAAGCTTGCTTCTCTGATGTTAGCGGCGGACGGGTGAGTAACACGTGGATAACCTACCTATAAGACTGGGATAACTTCGGGAAACCGGAGCTAATACCGGATAATATTTTGAACCGCATGGTTCAAAAGTGAAAGACGGTCTTGCTGTCACTTATAGATGGATCCGCGCTGCATTAGCTAGTTGGTAAGGTAACGGCTTACCAAGGCAACGATGCATAGCCGACCTGAGAGGGTGATCGGCCACACTGGAACTGAAACACGGTCCAG |
| ASV70 | GATGAACGCTGGCGGCGTGCCTAATACATGCAAGTCGAGCGAACGGACGAGAAGCTTGCTTCTCTGATGTTAGCGGCGGACGGGTGAGTAACACGTGGATAACCTACCTATAAGACTGGGATAACTTCGGGAAACCGGAGCTAATACCGGATAATATTTTGAACCGCATGGTTCAAAAGTGAAAGACGGTCTTGCTGTCACTTATAGATGGATCCGCGCTGCATTAGCTAGTTGGTAAGGTAACGGCTTACCAAGGCAACGATGCATAGCCGTCCTGAGAGGGTGATCGGCCACACTGGAACTGAGACACGGTCCAG |
| ASV1740 | TATGAACGCTGGCTTCTTTCCTCCTCCCTTCCCGTCGCGCGCCCGGCCGCGAAGCTTGCTTCTCTGATGTTAGCGGCGGACGGGTGAGTAACACGTGGATAACCTACCTATAAGACTGGGATAACTTCGGGAAACCGGAGCTAATACCGGATAATATTTTGAACCGCATGGTTCAAAAGTGAAAGACGGTCTTGCTGTCACTTATAGATGGATCCGCGCTGCATTAGCTAGTTGGTAAGGTAACGGCTTACCAAGGCAACGATGCATAGCCGACCTGAGAGGGTGATCGGCCACACTGGAACTGAGACACGGTCCAG |

**Table S2. Oligonucleotides used in the study.** Genotyping and Real Time qPCR primers are detailed. Titles describing information below are highlighted in bold.

| **Genotyping primer details** | | |
| --- | --- | --- |
| **Allele** | **Sequence 5'−3'** | |
| *JunB* - Fw | ATCCTCGTGGGAGCGGGGA | |
| *JunB* - Rw | AAACATACAAAATACGCTGG | |
| *S100a9* P1- Fw1 | TCAGCCGCTACAGTCAACAG | |
| *S100a9* P2 - Fw2 | GGTGGGGTATGACTGCAAGA | |
| *S100a9* P3 - Rw1 | AACTGATGGCGAGCTCAGAC | |
| *S100a9* P4 - Rw2 | ACAAATAGAAATGGAAACACCTTCT | |
| *S100a8* - Fw | CTAGCAGTGTCTAGCAGAAG | |
| *S100a8* - Rw | ACTGAAAGGAAAAGTCGCAG | |
| *Cre* A- Fw | CAATTTACTGACCGTACAC | |
| *Cre* B- Rw | CCCAGAAATGCCAGATTACG | |
| **Real Time qPCR primer details** | | |
| **Gene** | **Forward sequence 5'−3'** | **Reverse sequence 5'−3'** |
| *S100a8* | AAATCACCATGCCCTCTACAAG | CCCACTTTTATCACCATCGCAA |
| *S100a9* | ATACTCTAGGAAGGAAGGACACC | TCCATGATGTCATTTATGAGGGC |
| *Rlp4* | CTACTGCACTGGCAACCAAA | TCTTGGCAACCACCTTTTTC |

**Table S3. Antibodies and conditions used in the study.** Primary and secondary antibodies used for IHC, IF, flow cytometry and sorting are detailed. Titles describing information below are highlighted in bold.

| **Primary antibody details** | | | | |
| --- | --- | --- | --- | --- |
| **Target** | **Source** | **Reference** | **Application** | **Concentration** |
| CD16/32 | BD Pharmingen | 553142 | Flow cytometry | 1,25 μg/ml |
| CD11b-PerCP-Cy5 | BD Pharmingen | 550993 | Flow cytometry/ Sorting | 1 μg/ml |
| CD45-APC-Cy7 | BioLegend | 103116 | Flow cytometry/ Sorting | 1 μg/ml |
| Filaggrin | BioLegend | 905801 | IF | 1 μg/ml |
| Loricrin | BioLegend | 905101 | IF | 0,5 μg/ml |
| Gr-1 (Ly6-C and Ly6-G)-PE/Cyanine7 | BioLegend | 108416 | Flow cytometry | 1 μg/ml |
| Ki-67 | eBioscience | 14-5698-82 | IF | 2 μg/ml |
| K5 | BioLegend | 905501 | IF | 1 μg/ml |
| K10 | BioLegend | 905404 | IF | 1 μg/ml |
| K14 | BioLegend | 905301 | IF | 1 μg/ml |
| Ly6-C-APC | BD Pharmingen | 560595 | Flow cytometry/ Sorting | 1 μg/ml |
| Ly6-G-PE | BD Pharmingen | 551461 | Flow cytometry/ Sorting | 1 μg/ml |
| Ly6-G | BioLegend | 127601 | IF | 0,5 μg/ml |
| S100A8/Calgranulin A | Santa Cruz | sc-8113 | IHC/IF | 0,5 μg/ml |
| S100A9/Calgranulin B | Santa Cruz | sc-8115 | IHC/IF | 0,5 μg/ml |
| Calprotectin | R&D Systems | DY8596 | IHC/IF | 800 ng/ml |
| *S. aureus* | Abcam | ab20920 | IF | 5 μg/ml |
| **Secondary antibody details** | | | | |
| **Target** | **Source** | **Reference** | **Application** | **Conditions** |
| Alexa Fluor 488 donkey anti-goat | Thermo Fisher Scientific | A-11055 | IF | 1 μg/ml |
| Alexa Fluor 488 donkey anti-rabbit | Thermo Fisher Scientific | A-21206 | IF | 1 μg/ml |
| Alexa Fluor 555 donkey anti-rat | Thermo Fisher Scientific | A-78945 | IF | 1 μg/ml |
| Alexa Fluor 594 donkey anti-goat | Thermo Fisher Scientific | A-11058 | IF | 1 μg/ml |

**Table S4. ELISAs used in mouse skin and serum samples.** Titles describing information below are highlighted in bold.

| **ELISA details** | | |
| --- | --- | --- |
| **Target** | **Source** | **Reference** |
| Calprotectin | R&D Systems (DuoSet) | DY8596 |
| G-CSF | R&D Systems (DuoSet) | DY414 |
| IgE | Immunology Consultants Laboratory | E-90E |
| IL-17A | R&D Systems (DuoSet) | DY421 |
| IL-36β | R&D Systems (DuoSet) | DY2298 |
| IL-6 | R&D Systems (Quantikinet) | M6000B |
| MPO | R&D Systems (DuoSet) | DY3667 |
| NE | R&D Systems (DuoSet) | DY4517 |
| S100A8 | R&D Systems (DuoSet) | DY3059 |
| S100A9 | R&D Systems (DuoSet) | DY2065 |

**Supplementary Figure legends**

**Fig. S1. Increased *A8* and *A9* expression in epidermal and myeloid cells in human and murine skin inflammation. a** UMAP of scRNA-seq analysis of 46,917 CD45^+^ enriched human skin cells, colored by sample type. **b** Cell types identified by hierarchical clustering in human skin. **c** Number of cells belonging to each sample and cell type (top) in humans and expression levels of *A8* and *A9* by sample and cell type (bottom). **d** UMAP of scRNA-seq analysis of skin from BALB/c mice, mock-treated or topically infected with SA alone for 48h or MC903-treated for 14 days followed by topical SA infection. 18,790 total cells identified, colored by sample. **e** Mouse cell types identified by hierarchical clustering. **f** Number of cells belonging to each sample and cell type (top) in mice and expression levels of *A8* and *A9* by mouse sample and cell type (bottom). **g** Representative IHC images of A8 (top) and A9 (bottom) in *JunB^∆ep^* mice at different ages, from 2 months of age before disease onset to 8 months of age, after development of skin lesions at 6 months-old. Scale bars, 100μm. **h** Protein levels of A8, A9 and CP in the serum of 2 months-old *JunB^∆ep^* mice and control littermates. Dot plots represent mean ± SEM. ***p* ≤ 0.01. Unpaired 2-tailed Student’s t-test with Welch’s correction was applied to compare statistical difference between the groups.

**Fig. S2. Genetic inactivation of *A9* in epidermal cells aggravates skin disease in *JunB^∆ep^* mice. a** Representative H&E pictures of the facial and ventral skin in the indicated mice. Scale bars, 100μm. **b** Flow cytometric analysis of CD45^+^ immune cells in the skin of control, *JunB^∆ep^* and *JunB^∆ep^S100a9^∆ep^* mice, shown as percentage (%) of live cells. **c** Representative IF images of A8 (red; top panel), A9 (red, middle panel) and CP (red; bottom panel) co-stained with K5 (green) in the skin of *JunB^∆ep^* and *JunB^∆ep^S100a9^∆ep^* mice. Nuclei are stained with DAPI. White arrows point to A8-, A9- or CP-positive cells. Scale bars, 200μm. **d** *A8* and *A9* gene expression analysis of neutrophils sorted from skin lysates of *JunB^∆ep^* and *JunB^∆ep^S100a9^∆ep^* mice. **e** Body weight in grams (g) (left) and spleen to body weight ratio (right) in indicated mice. **f** Flow cytometric analysis of bone marrow CD45^+^ immune cells (left), shown as percentage (%) of live cells, and bone marrow neutrophils (CD11b^+^, Ly6C^+^, Ly6G^+^) (right), shown as percentage (%) of CD45^+^ cells in control, *JunB^∆ep^* and *JunB^∆ep^S100a9^∆ep^* mice. **g** Lymphocyte counts in the blood of control, *JunB^∆ep^* and *JunB^∆ep^S100a9^∆ep^* mice. Dot plots represent mean ± SEM. **p* ≤ 0.05, ***p* ≤ 0.01, ****p* ≤ 0.001, *****p* ≤ 0.0001. One-way ANOVA with Fishers’ LSD test was used for statistical grouped analysis.

**Fig. S3. Loss of A9 in neutrophils ameliorates skin inflammation in *JunB^∆ep^* mice. a** Schematic diagram for genetic Cre/LoxP inactivation of *A9* using the myeloid/granulocyte-specific MRP8-Cre-ires/GFP. **b** Representative gating strategy for flow cytometry showing GFP^+^ neutrophils in the blood of *A9^∆n^* mice (top) and GFP^+^ neutrophils in bone marrow, spleen and blood of control and *A9^∆n^* mice (bottom). **c** *A9* deletion efficiency in GFP^+^ sorted cells of 3 *A9^∆n^* mice (indicated as 1, 2, 3), compared to GFP^-^ sorted cells. **d** Flow cytometric analysis of bone marrow (BM) neutrophils (CD11b^+^, Ly6C^+^, Ly6G^+^), shown as percentage (%) of CD45^+^ cells in control and *A9^∆n^* mice. **e** Body weight in grams (g) (left) and spleen-to-body weight ratio (%) (right) in control and *A9^∆n^* littermates. **f** White blood cell (WBC) counts, blood granulocytes (total counts, left, and % of WBC, right) and lymphocytes (total counts, left, and % of WBC, right) in control and *A9^∆n^* mice. **g** Representative IF images of A9-positive cells (red) co-stained with K5 (green), A8-positive cells (red), as well as A8-positive cells (green) and neutrophils (red) in control::*JunB^∆ep^* and *A9^∆n^::JunB^∆ep^* skin sections. Nuclei are stained with DAPI. Epidermis and dermis are divided by a white line. Scale bars, 200μm. **h** Body weight in grams (g) (left) and spleen-to-body weight ratio (right) in control::*JunB^∆ep^* and *A9^∆n^::JunB^∆ep^* mice. Dot plots represent mean ± SEM. Heat map represents mean normalized to the maximum value within each group and scaled by row, **p* ≤ 0.05. Unpaired 2-tailed Student’s t-test with Welch’s correction was applied to compare statistical difference between the groups.

**Fig. S4. *A9* knock-out improves skin inflammation, but aggravates systemic disease in *JunB^∆ep^* mice. a** Representative H&E and IF images of K5-positive (K5^+^) keratinocytes (red) in the snout (top 2 panels) and ventral skin (bottom 2 panels) of control, *S100a9^-/-^*, *JunB^∆ep^* and *JunB^∆ep^S100a9^-/-^* mice. Nuclei are stained with DAPI. Scale bars, 100μm. **b** SA CFUs in the ventral skin in indicated mice. **c** Representative IF images of A8 and A9 in the snout of control, *S100a9^-/-^*,  *JunB^∆ep^* and *JunB^∆ep^S100a9^-/-^* mice. Nuclei are stained with DAPI. Scale bars, 100μm. White line divides epidermis and dermis. **d** Flow cytometric analysis of CD45^+^ cells in the skin of control, *S100a9^-/-^, JunB^∆ep^* and *JunB^∆ep^S100a9^-/-^* mice, shown as percentage (%) of live cells. **e** Body weight in grams (g) (left) and spleen-to-body weight ratio (right) in control, *S100a9^-/-^*, *JunB^∆ep^* and *JunB^∆ep^S100a9^-/-^* mice. **f** Flow cytometric analysis of bone marrow CD45^+^ immune cells (left), shown as percentage (%) of live cells, and bone marrow neutrophils (CD11b^+^, Ly6C^+^, Ly6G^+^) (right), shown as percentage (%) of CD45^+^ cells in bone marrow from control, *S100a9^-/-^*, *JunB^∆ep^* and *JunB^∆ep^S100a9^-/-^* mice. **g** Blood lymphocyte counts in indicated mice. Dot plots represent mean ± SEM. **p* ≤ 0.05, ***p* ≤ 0.01, ****p* ≤ 0.001, *****p* ≤ 0.0001. One-way ANOVA with Fishers’ LSD test was used for statistical grouped analysis.

**Fig. S5. Digit swelling and local bone destruction with SA colonization in *JunB^∆ep^S100a9^-/-^* mice**. **a** Representative H&E and images of control, *S100a9^-/-^*, *JunB^∆ep^* and *JunB^∆ep^S100a9^-/-^* digits. Black arrow indicates epidermal thickening. Scale bar, 200μm. **b** Bone damage score in indicated mice. **c** Representative images of TRAP staining in control, *JunB^∆ep^*, *S100a9^-/-^* and *JunB^∆ep^S100a9^-/-^* digit sections. Black arrow indicates osteoclasts in *JunB^∆ep^S100a9^-/-^* mice. Scale bar, 100μm. **d** 16S rRNA sequencing analysis of SA single colonies isolated from the face and ventral skin of *JunB^∆ep^* and *JunB^∆ep^ S100a9^-/-^* mice, as well as the digits of *JunB^∆ep^* *S100a9^-/-^* mice. **e** SA CFUs in the distal phalanges of control, *JunB^∆ep^*, *S100a9^-/-^* and *JunB^∆ep^S100a9^-/-^* mice. **f** *A8* expression in the skin of the digits in all 4 analyzed groups. **g** *A8* gene expression in neutrophils sorted from the skin of the digits in indicated mice. **h** Scheme of study layout for bone marrow transplantation (BMT) experiment. **i** Representative pictures of *control::JunB^∆ep^S100a9^∆ep^* and *A9^∆n^:: JunB^∆ep^S100a9^∆ep^* bone marrow chimeric mice showing the snout (top panel) and digits (bottom panel). **j** SA CFUs in the skin of the snout and digits in indicated mice. **k** Percentage of CD45^+^ neutrophils (CD11b^+^, Ly6C^+^, Ly6G^+^) in the skin from the snout or digits of the indicated bone marrow chimeras. **l** Percentage of CD45^+^ neutrophils (CD11b^+^, Ly6C^+^, Ly6G^+^) in the BM of the 2 indicated groups of mice. **m** Body weight in grams (g) (left) and spleen-to-body weight ratio (%) (right) of *control::JunB^∆ep^S100a9^∆ep^* and *A9^∆n^::JunB^∆ep^S100a9^∆ep^* mice. Dot plots represent mean ± SEM. **p* ≤ 0.05, ***p* ≤ 0.01, ****p* ≤ 0.001, *****p* ≤ 0.0001. One-way ANOVA with Fishers’ LSD test was used for statistical grouped analysis and unpaired 2-tailed Student’s t-test with Welch’s correction was applied to compare statistical difference between 2 groups (grey stars).

**Fig. S6. Loss of *A8* in epidermal cells improves skin and systemic disease in *JunB^∆ep^* mice. a** Representative H&E images (top panel) and IF pictures of filaggrin (red) and loricrin (red) co-stained with K5 (green) in ventral skin of control, *JunB^∆ep^* and *JunB^∆ep^S100a8^∆ep^* mice. Nuclei are stained with DAPI. Scale bars, 200μm. **b** Flow cytometric analysis of CD45^+^ immune cells in the skin of control, *JunB^∆ep^* and *JunB^∆ep^S100a8^∆ep^* mice, shown as percentage (%) of live cells. **c** Body weight in grams (g) (left) and spleen-to-body weight ratio (right) of control, *JunB^∆ep^* and *JunB^∆ep^S100a8^∆ep^* mice. **d** Flow cytometric analysis of bone marrow CD45^+^ immune cells and bone marrow neutrophils (CD11b^+^, Ly6C^+^ and Ly6G^+^) in indicated mice, shown as percentage (%) of CD45^+^ cells. **e** Flow cytometric analysis of blood neutrophils (CD11b^+^, Ly6C^+^ and Ly6G^+^) in indicated mice, shown as percentage (%) of CD45^+^ cells. **f** Blood lymphocyte counts in the indicated groups. **g** SA CFUs in the distal phalanges of control, *JunB^∆ep^*, *JunB^∆ep^S100a8^∆ep^* mice. Dot plots represent mean ± SEM. **p* ≤ 0.05, ***p* ≤ 0.01, ****p* ≤ 0.001, *****p* ≤ 0.0001. One-way ANOVA with Fishers’ LSD test was used for statistical grouped analysis and unpaired 2-tailed Student’s t-test with Welch’s correction was applied to compare statistical difference between 2 groups (grey stars).

**Supplementary Methods**

**Mice**

The following C57BL/6 genetically engineered mouse models were used in this study: *S100a8*^flox/flox^, *S100a9*^flox/flox^, Mrp8-Cre^Tg^, *JunB^∆ep^, S100a9^∆ep^, JunB^∆ep^S100a9^∆ep^, A9^∆n^, S100a9^-/-^, JunB^∆ep^S100a9^-/-^S100a8^∆ep^* and *JunB^∆ep^S100a8^∆ep^. JunB*^flox/flox^ allele and *JunB^∆ep^* mice were previously described (1). *S100a9* knock-out mice, *S100a9* floxed mice with loxP sites around exon 3 and S100a8 floxed mice with loxP sites around exon 2 were previously described (2, 3). *S100a9* and *S100a8* floxed mice, as well as *S100a9^-/-^* mice were crossed with *JunB^∆ep^* to generate *JunB^∆ep^S100a9^∆ep^ JunB^∆ep^S100a8^∆ep^* and *JunB^∆ep^S100a9^-/-^* mice, respectively. *S100a8* floxed mice were obtained from Johannes Roth and Sandra Bachg (University of Münster). Mrp8-Cre^Tg^ mice previously described(4) were obtained from Sylvia Knapp (MUV). Primer sequences used for routine genotyping are listed in Table S2. Matings were set up with one parent hemizygous for the K5-Cre^Tg^ allele. Mice were co-housed (4-8 males or 4-10 females) in Specific Pathogen-Free conditions at the Animal Facility of the MUV in controlled environment with unrestricted access to water and standard chow. All mouse experiments were approved under license under license 66.009/171-V/3b/2018, and conducted according to local/institutional policies and national and European regulations. Unless otherwise stated, age-matched 6-months old mice were used for the experiments and both sexes were included, since no gender differences were observed.

**Bone marrow transplantation (BMT)**

Bone marrow-derived 5×10^6^ control or *A9^∆n^* cells in 100μl sterile PBS were injected into tail vein of 12 Gy (2 doses of 6Gy 𝛄-irradiation) lethally irradiated *JunB^∆ep^* or *JunB^∆ep^S100a9^∆ep^* recipients. A minimum of 2 control mice were irradiated in parallel to secure that the irradiation was lethal. Female donors were employed, and a total of 3 independent experiments were conducted as part of the BMT study.

**Macroscopic skin inflammation scoring**

Skin inflammation was scored based on macroscopic skin lesions in the face and/or ventral skin of the mouse from mild to severe as follows: no phenotype (0), mild with loss of hair in one region (1), mild with loss of hair in both regions (2), moderate with lesions in one region (3) and severe with lesions in both regions (4).

**Bacterial sampling and 16S rRNA gene sequencing and bioinformatics**

Sterile cotton swabs (Lohmann & Rauscher) were moistened in sterile PBS prior to probing the skin of mice, followed by dissolving in PBS. After centrifugation (5min at 3000g), pellets were resuspended in 100µl and serial dilutions were plated in triplicates on Baird-Parker agar (Sigma) plates. After overnight-incubation at 37°C, colonies were counted and bacterial load (colony forming units; CFUs) was calculated. For identification of bacteria, single colonies were picked from Baird-Parker Agar plates and inoculate into1ml tryptic soy broth (Sigma). After overnight incubation (37°C, 180rpm shaking), 500µl of bacterial suspensions were centrifuged (5min at 3000g) and pellets were stored at -20°C until further processing. For 16S RNA sequencing, bacterial DNA was isolated and processed as previously described(5) with minor modifications. Briefly, DNA was isolated using the DNeasy PowerSoil Pro Kit (Qiagen). The V1-V2 hyper variable region of the 16S rRNA gene was pre-amplified for 10 PCR cycles using Illumina adaptor-linked PCR primers (5, 6) and the Accuprime Taq DNA Polymerase High Fidelity kit (Invitrogen). One tenth volume of pre-amplified DNA was used as template for an enrichment PCR (same parameters/primers) for another 30 cycles. Following analysis and quantification of PCR products by automated electrophoresis using the 4200 TapeStation system (Agilent), libraries containing pooled equimolar PCR products were purified using AMPure XP beads (Beckman Coulter), spiked with 40% phiX (Illumina) and sequenced using Illumina MiSeq technology (2x 350 bp configuration; MiSeq Reagent kit v3) at the Biomedical Sequencing Facility of CeMM and the MUV. 16S rRNA gene reads were annotated using dada2 (7)] pipeline (v1.20.0) in R. Reads were trimmed to 225 base pairs, filtered and taxonomy assignment was performed with assignTaxonomy and addSpecies employing Silva reference database (8) v138.1 with default parameter settings. Amplicon sequence variant (ASV) reads were further analyzed using nucleotide BLAST (<https://blast.ncbi.nlm.nih.gov>, Database: rRNA/ITS databases, 16S ribosomal RNA sequences [Bacteria and Arachaea]). Identified species with highest identity (%) were assigned to ASVs (Table S1).

**Micro-computed tomography imaging (μCT)**

Bone parameters were assessed in the tibiae and in the digits of hind limbs. Female mice were selected, to avoid differences in bone structures that may arise from sex dimorphism. Samples previously fixed in 7.5% formalin-PBS for a minimum of 48h and stored in 70% ethanol-PBS, were scanned using μCT35 (Scanco Medical) with settings of 55kVp, 145μA, 8 W, 300 ms, and high resolution. Trabecular bone variables were assessed in 350 slices of the proximal tibiae. Cortical bone variables were evaluated in the cortex of the tibial mid-shaft (200 slices). Reconstruction thresholds of 280 (trabecular bone) or 310 (cortical bone) were used as previously described (9).

**Histology**

Tissues were fixed in PBS, 7.5% formalin. Hind-limb paws were decalcified after 48h of fixation, with 18% EDTA in PBS (pH 8.0) for a minimum of 2 weeks. Paraffin was removed from 5µm thick sections using xylene and bathed in decreasing alcohol concentrations (100%-96%-70%-50%-30% ethanol) followed by water and PBS washes. Sections were stained with Hematoxylin and eosin. Epidermal thickness assessment was performed using Image J, by analyzing 4 different measurements for each skin section of the mice. Digits were also subjected to tartrate-resistant acid phosphatase (TRAP) staining (Sigma) as previously described(10), to determine number of TRAP^+^ multinucleated osteoclasts (more than 3 nuclei). Immunohistochemistry (IHC) and immunofluorescence (IF) were performed following antigen retrieval using pH=6 citrate buffer in either a pressure cooker for skin samples or microwave for paws. Non-specific binding was blocked with 10% fetal bovine serum or 1% bovine serum albumin in PBS-T (0.1% of Tween20) for about 30min. Primary antibodies (Table S3) were incubated overnight at 4°C. For IHC biotin/streptavidin amplification and HRP-based chromogen detection (Vectastain ABC Kit or and DAB; Vector Laboratories, Inc) were used following manufacturer´s instructions. For IF, secondary antibodies conjugated with Alexa Fluor^®^ 488, 555 or 594 were used for 1h at room temperature. Bright-field or fluorescence BX63 Olympus was used for IHC and IF, respectively.

**Flow cytometry and cell sorting**

Skin, spleen, blood and bone marrow were analyzed by flow cytometry and/or sorting as indicated. Skin was cut in small pieces and subjected to digestion with liberaseTM (Roche) 1h at 37°C shaking mildly. Single-cell suspensions were filtered into 70µm cell-strainer and blocked with anti-CD16/32 antibody (BioLegend). Subsequently, cells were stained with cell-surface antibodies (Table S3) for 30min at 4°C. After incubation, cells were washed, filtered into 70µm cell-strainer, and stained with 7-AAD Viability Staining Solution (BioLegend) for Flow cytometric analysis or with DAPI for cell sorting, according to the manufactures protocol to exclude dead cells. Samples were collected in a LSR Fortessa cell analyzer (BD Biosciences) or in FACSAria Cell Sorter (BD Biosciences). At least 50 000 single events were collected in flow cytometry experiments. Around 100 000 cells were sorted from skin samples and 300 000 cells from the bone marrow of control and *A9^∆n^* mice. Data were analyzed using FlowJo software (version 10.8.1, Treestar). Dead cells and doublets were excluded for cell-sorting procedure. Neutrophils were purified into TRItidy G reagent (Panreac AppliChem) for RNA isolation. The antibodies and conditions used for sorting are detailed in Table S3.

**White Blood Cell (WBC) Count**

Blood was collected by cardiac puncture with 25G needles and collected in EDTA tubes. 10μl were used to count white blood cells in Scil Vet abc Plus™ hematology analyser (Scil Animal Care Company), including granulocytes and lymphocytes.

**RNA isolation and qPCR**

The RNA was isolated using TRItidy G (Panreac AppliChem), and complementary DNA was synthesized using GoScript™ Reverse Transcription Mix, Oligo(dT) (Promega) and for RT qPCR GoTaq qPCR Master Mix (Promega) and Bio-Rad fluorescence thermocyclers were used, all according to the manufacturer’s instructions. Relative quantification of target genes was performed according to the 2^-ΔΔCt^ method. Expression levels of the genes of interest were normalized using at least one housekeeping gene. Primer sequences are listed in Table S3.

**scRNAseq data analysis**

**Human scRNAseq:** Human data was downloaded from GEO database (GSE153760) (11)] Details of sample collection, processing, library generation, sequencing, and counting with Cell Ranger are described by Rojahn *et al* (11). Count matrices were developed in R for the creation of a Seurat object. Cells with <50 features, >5000 features, >22000 counts, or >15% mitochondrial genes were removed. Data was normalized and 2000 variable features were identified for use in scaling the data and performing linear dimensional reduction with RunPCA and standard parameters. Clusters were identified with FindNeighbors (30 PCs) and FindClusters functions with a range of resolutions. For each resolution, visualization was performed with the RunUMAP function (30 PCs) and marker genes for each cluster were determined with the FindAllMarkers function with min.pct = 0.25. The resolution yielding clusters with the most distinct marker genes was chosen for further analysis (0.8). Cell type annotations were assigned based on the expression of marker genes.

**Mouse scRNAseq**: For mouse cell-specific A8 and A9 expression analysis, dscRNAseq data was downloaded from DDBJ sequence Read Archive (www.ddbj.nig.ac.jp), accession number DRA015287 (12) Details of sample collection, processing, library generation, sequencing, counting with Cell Ranger, and creation of a Seurat object were described by Nakatsuji *et al.*(12). 50 PCs were chosen for hierarchical clustering, with a resolution of 0.1. Cell type annotations were assigned based on the expression or marker genes, using the FindAllMarkers function with min.pct = 0.25.

**Protein isolation**

Skin samples (approximately 5 x 5mm) from the face (snout), ventral skin or surrounding the digits were weight, cut and snap-frozen in liquid nitrogen immediately after sacrificing the mice. Samples were then transferred into new 1.5ml microcentrifuge tubes containing 500µl RIPA buffer (1.6mM NaH_2_PO_4_, 8.4mM Na_2_HPO_4,_ 0.1% SDS, 100mM NaCl, 0.1% Triton X100, 0.5% sodium deoxycholate in distilled water) and 1x protease inhibitor cocktail (Roche) followed by tissue homogenization in Precellys 24. Protein content was measured using the micro BCA protein assay Kit (Thermo Scientific). 30μg of total protein were used per skin sample for subsequent analysis.

**Analysis of inflammatory mediators by enzyme-linked immunosorbent assay (ELISA)**

Serum was collected after centrifugation of the blood in tubes with a polyester gel. Serum and skin protein lysates (30µg of total protein) were analyzed using commercial ELISA kits (Table S4) following manufacturer's instructions. Unless otherwise specified, snout was used for skin ELISA experiments. Protein levels were measured in ng/ml.

**Generation of graphs and graphical abstract**

Graphs were generated using GraphPad Prism software version 10.3.0 for Windows and Mac OS (GraphPad Software, La Jolla, CA, USA). Bar plots from 16S rRNA sequencing (Fig. S5D) were generated using the R package Microbiome by Lahti, Shetty *et al.* (<http://microbiome.github.io>). Graphical schemes were created using Smart Servier Medical Art by Servier, licensed under a Creative Commons Attribution 3.0 Unported License (https://smart.servier.com/) and BioRender (<https://www.biorender.com/>).

**Statistical analysis**

Statistical analysis of the results was performed using GraphPad Prism 10 (GraphPad Software, La Jolla, CA, USA). Normal distribution of data was assessed and statistical significance was determined by one-way ANOVA with Fishers’ LSD test for grouped analysis and unpaired 2-tailed Student’s t-test with Welch’s correction was applied to compare statistical difference between 2 groups. Data are shown in dot plots with the mean and error bars denote the standard error of the mean (SEM). Each dot represents a single mouse, unless otherwise stated. ELISA results are displayed as heat maps, representing the normalized mean values (C/Cmax) for each experimental group. Statistical significance was defined as p value equal to or below 0.05, and indicated as **p* ≤ 0.05 or •*p* ≤ 0.05 or #*p* ≤ 0.05. Grey stars indicate significant difference between 2 subgroups within the entire tested group, as calculated by unpaired 2-tailed Student’s t-test.

**Supplementary References**

1. Meixner A, Zenz R, Schonthaler HB, Kenner L, Scheuch H, Penninger JM, Wagner EF. Epidermal JunB represses G-CSF transcription and affects haematopoiesis and bone formation. Nat Cell Biol. 2008;10(8):1003-11.

2. Mellor LF, Gago-Lopez N, Bakiri L, Schmidt FN, Busse B, Rauber S, et al. Keratinocyte-derived S100A9 modulates neutrophil infiltration and affects psoriasis-like skin and joint disease. Ann Rheum Dis. 2022;81(10):1400-8.

3. Skryabin BV, Kummerfeld DM, Gubar L, Seeger B, Kaiser H, Stegemann A, et al. Pervasive head-to-tail insertions of DNA templates mask desired CRISPR-Cas9-mediated genome editing events. Sci Adv. 2020;6(7):eaax2941.

4. Passegue E, Wagner EF, Weissman IL. JunB deficiency leads to a myeloproliferative disorder arising from hematopoietic stem cells. Cell. 2004;119(3):431-43.

5. Watzenboeck ML, Drobits B, Zahalka S, Gorki AD, Farhat A, Quattrone F, et al. Lipocalin 2 modulates dendritic cell activity and shapes immunity to influenza in a microbiome dependent manner. PLoS Pathog. 2021;17(4):e1009487.

6. Rapin A, Pattaroni C, Marsland BJ, Harris NL. Microbiota Analysis Using an Illumina MiSeq Platform to Sequence 16S rRNA Genes. Curr Protoc Mouse Biol. 2017;7(2):100-29.

7. Callahan BJ, McMurdie PJ, Rosen MJ, Han AW, Johnson AJ, Holmes SP. DADA2: High-resolution sample inference from Illumina amplicon data. Nat Methods. 2016;13(7):581-3.

8. Quast C, Pruesse E, Yilmaz P, Gerken J, Schweer T, Yarza P, et al. The SILVA ribosomal RNA gene database project: improved data processing and web-based tools. Nucleic Acids Res. 2013;41(Database issue):D590-6.

9. Hayer S, Niederreiter B, Kalkgruber M, Wanic K, Maissner J, Smolen JS, et al. Analysis of combined deficiency of interleukin-1 and -6 versus single deficiencies in TNF-mediated arthritis and systemic bone loss. Bone Joint Res. 2022;11(7):484-93.

10. Hayer S, Vervoordeldonk MJ, Denis MC, Armaka M, Hoffmann M, Backlund J, et al. 'SMASH' recommendations for standardised microscopic arthritis scoring of histological sections from inflammatory arthritis animal models. Ann Rheum Dis. 2021;80(6):714-26.

11. Rojahn TB, Vorstandlechner V, Krausgruber T, Bauer WM, Alkon N, Bangert C, et al. Single-cell transcriptomics combined with interstitial fluid proteomics defines cell type-specific immune regulation in atopic dermatitis. J Allergy Clin Immunol. 2020;146(5):1056-69.

12. Nakatsuji T, Brinton SL, Cavagnero KJ, O'Neill AM, Chen Y, Dokoshi T, et al. Competition between skin antimicrobial peptides and commensal bacteria in type 2 inflammation enables survival of S. aureus. Cell Rep. 2023;42(5):112494.
