# Supplementary figures and images for: Chronic skin and systemic inflammation modulated by S100A8 and S100A9 complexes
Source: Cell Death Differ. 2025 Apr 11;32(10):1833–44. doi: 10.1038/s41418-025-01504-9 (PMC12501315; doi:10.1038/s41418-025-01504-9)

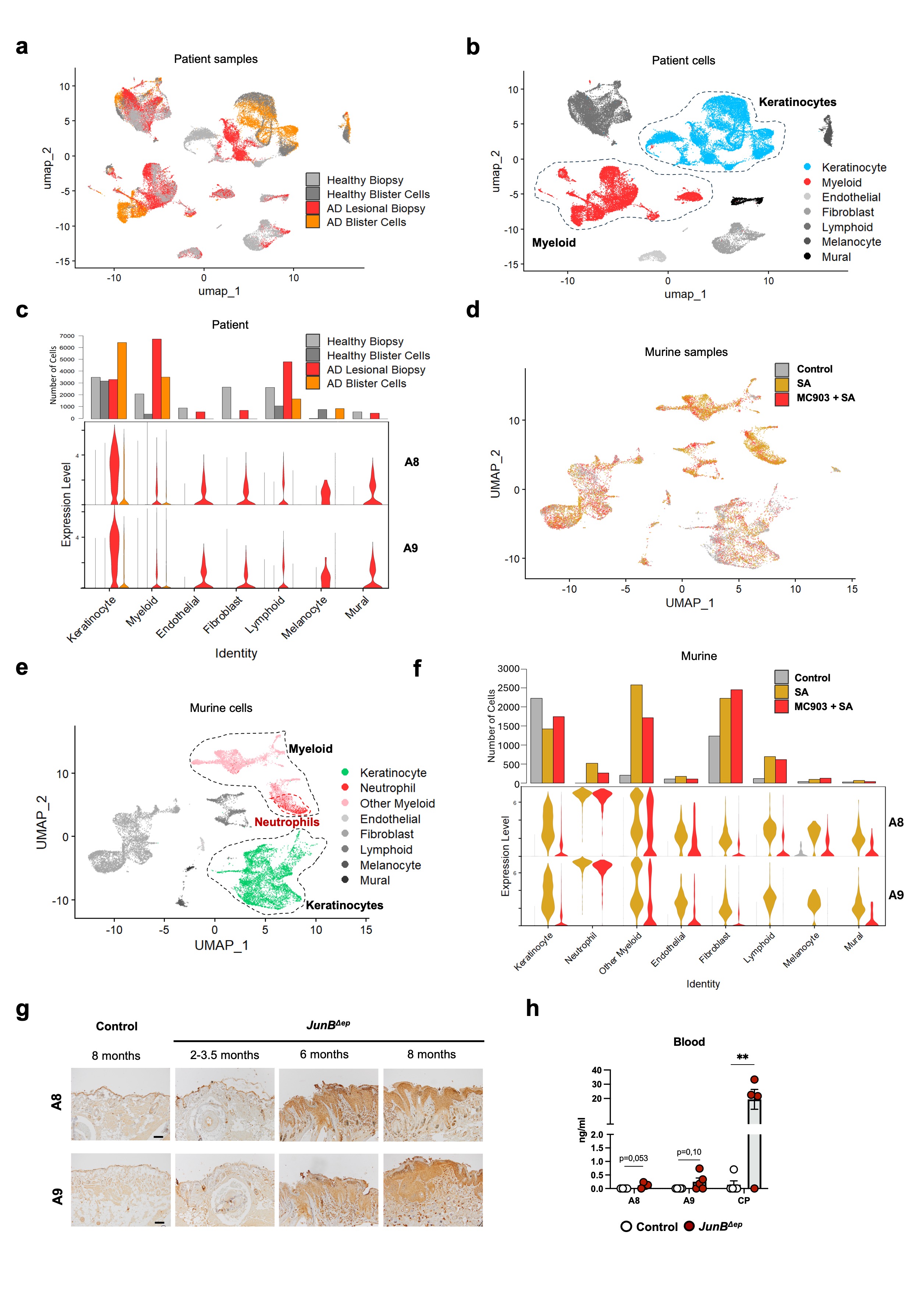

Supplement: Supplementary file 2 — Figure S1 [file 41418_2025_1504_MOESM2_ESM.jpg]

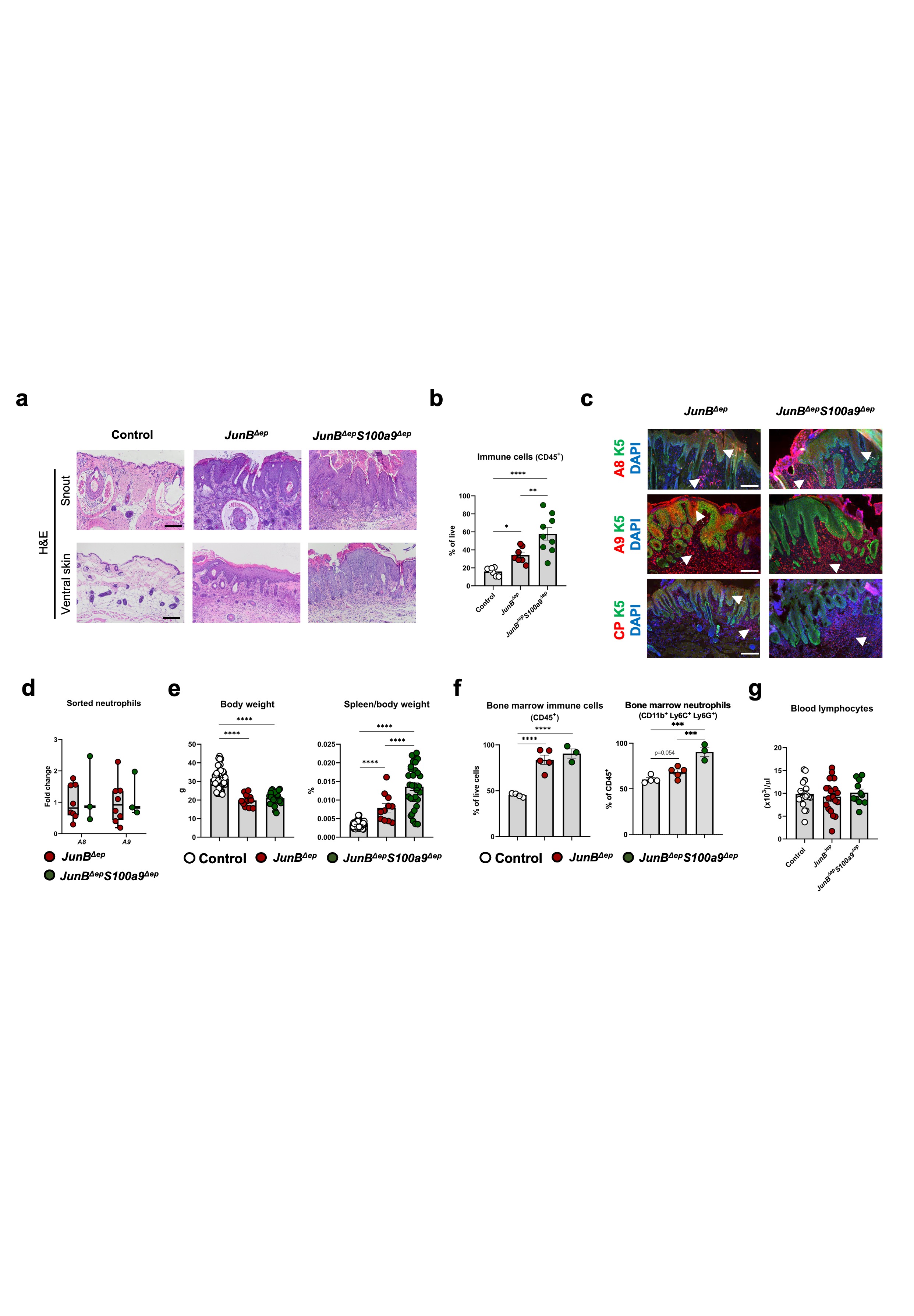

Supplement: Supplementary file 3 — Figure S2 [file 41418_2025_1504_MOESM3_ESM.jpg]

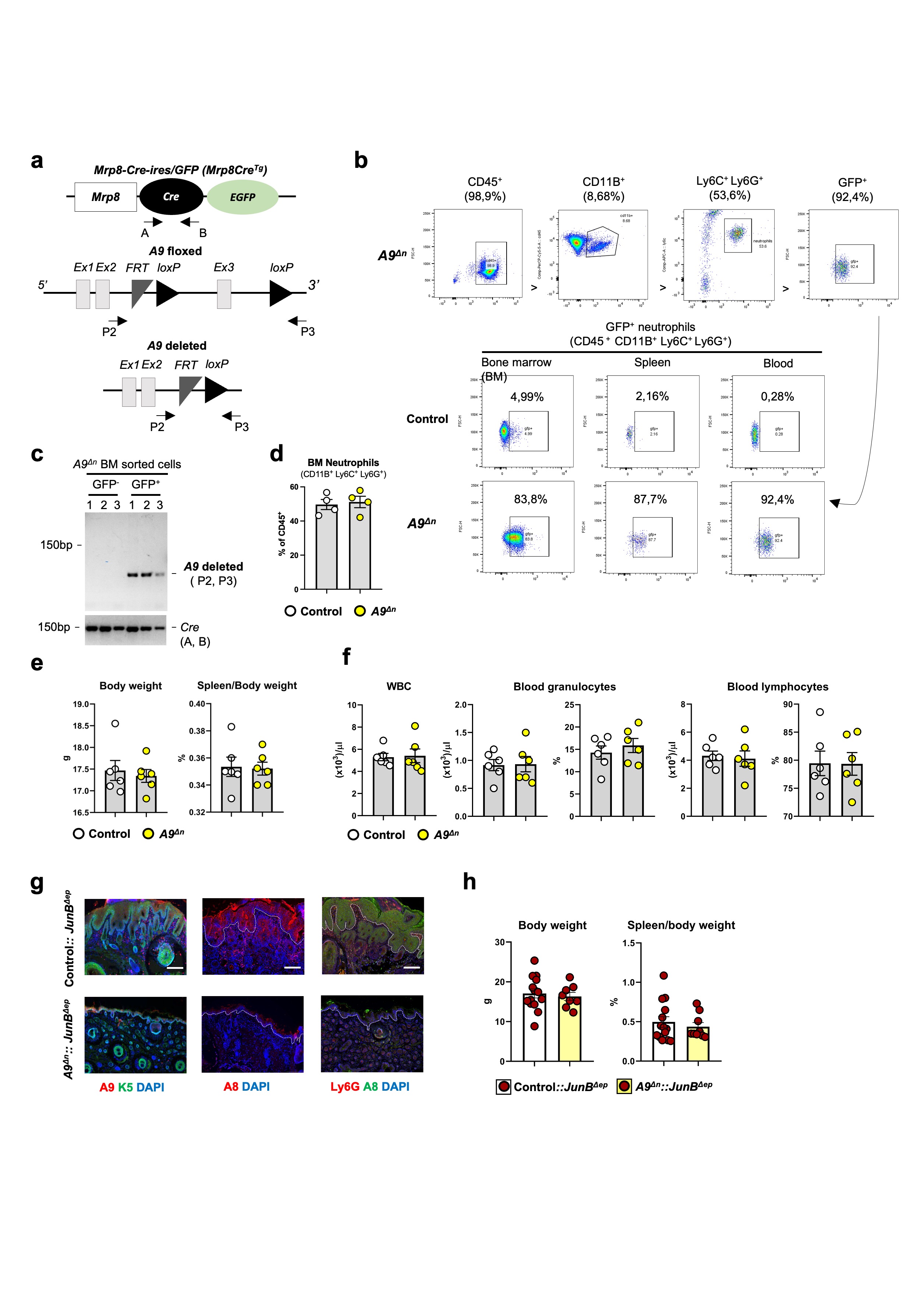

Supplement: Supplementary file 4 — Figure S3 [file 41418_2025_1504_MOESM4_ESM.jpg]

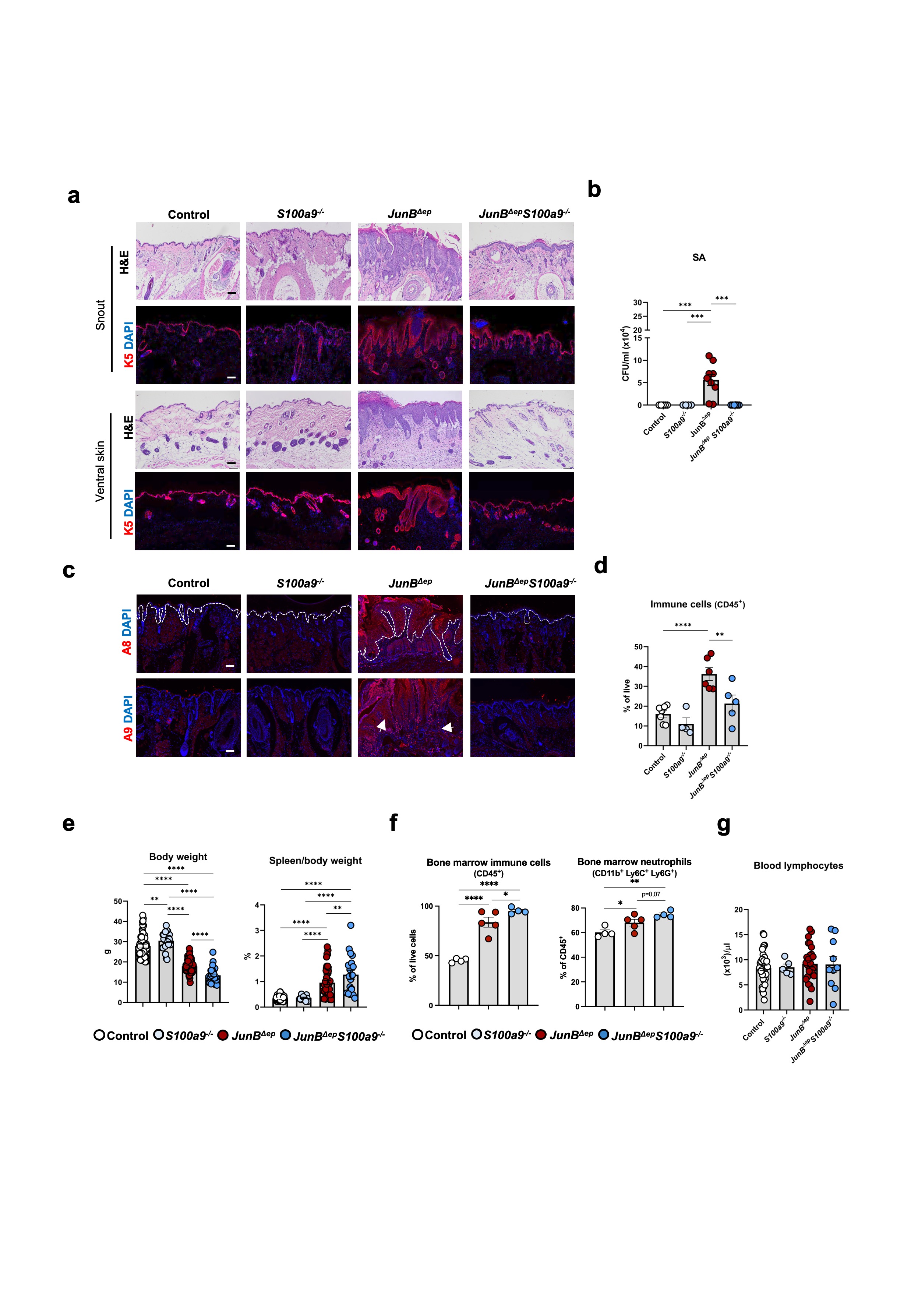

Supplement: Supplementary file 5 — Figure S4 [file 41418_2025_1504_MOESM5_ESM.jpg]

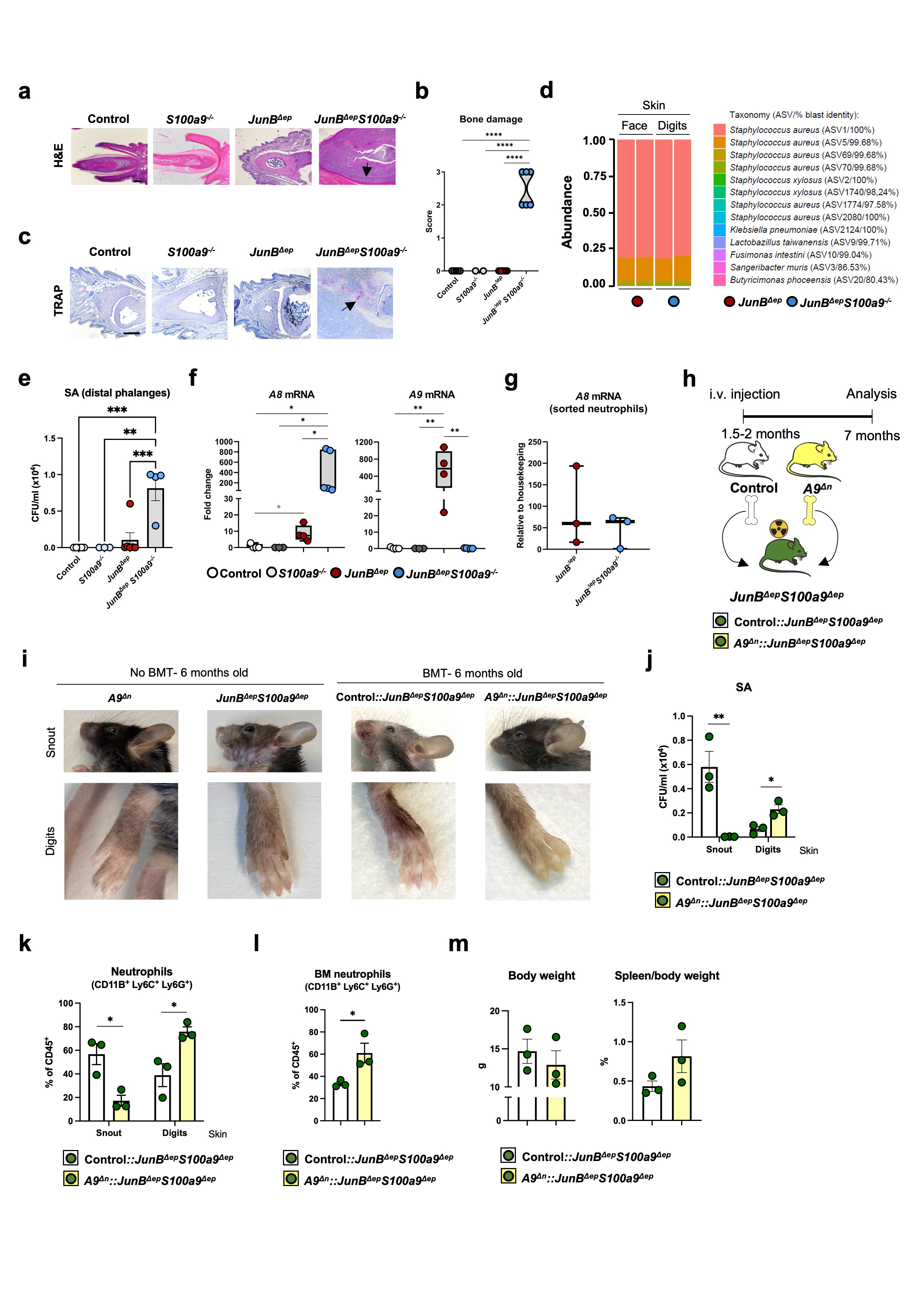

Supplement: Supplementary file 6 — Figure S5 [file 41418_2025_1504_MOESM6_ESM.jpg]

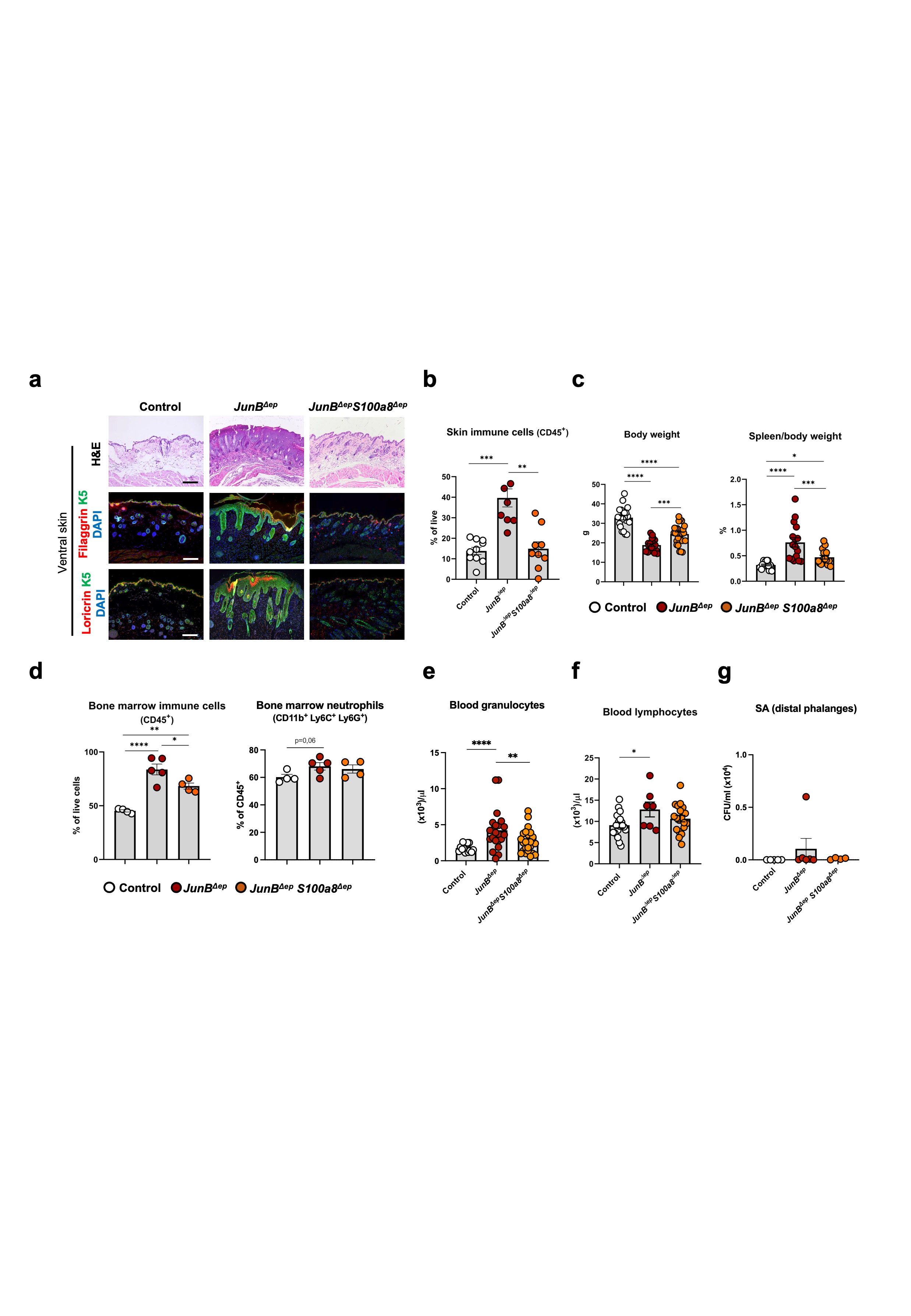

Supplement: Supplementary file 7 — Figure S6 [file 41418_2025_1504_MOESM7_ESM.jpg]
